# Molecular modification enables CO_2_ electroreduction to methane on platinum surface in acidic media

**DOI:** 10.1093/nsr/nwae361

**Published:** 2024-11-19

**Authors:** Hengpan Yang, Huizhu Cai, Deliang Li, Yan Kong, Shangzhao Feng, Xingxing Jiang, Qi Hu, Chuanxin He

**Affiliations:** College of Chemistry and Environmental Engineering, Shenzhen University, Shenzhen 518060, China; College of Chemistry and Environmental Engineering, Shenzhen University, Shenzhen 518060, China; College of Chemistry and Environmental Engineering, Shenzhen University, Shenzhen 518060, China; College of Chemistry and Environmental Engineering, Shenzhen University, Shenzhen 518060, China; College of Chemistry and Environmental Engineering, Shenzhen University, Shenzhen 518060, China; College of Chemistry and Environmental Engineering, Shenzhen University, Shenzhen 518060, China; College of Chemistry and Environmental Engineering, Shenzhen University, Shenzhen 518060, China; College of Chemistry and Environmental Engineering, Shenzhen University, Shenzhen 518060, China

**Keywords:** CO_2_ electroreduction, platinum catalyst, molecular modification, acidic media, methane product

## Abstract

Cu-based materials can produce hydrocarbons in CO_2_ electroreduction (CO_2_RR), but their stability still needs to be enhanced particularly in acidic media. Metallic Pt is highly stable in both acidic and alkaline media, yet rarely utilized in CO_2_RR, due to the competitive activity in hydrogen evolution reaction (HER). In this research, abundant thionine (Th) molecules are stably confined within Pt nanocrystals via a molecular doping strategy. The Pt surface is successfully modulated by these Th molecules, and thereby the dominant HER activity is converted to CO_2_RR activity. CO_2_ could be electroreduced to CH_4_ using organic molecule-modified Pt-based catalysts for the first time. Specifically, this composite catalyst maintains more than 100-hour stability in strong acid conditions (pH 1), even comparable to those state-of-the-art CO_2_RR catalysts. *In-situ* spectroscopic analysis and theoretical calculations reveal that the molecular modification can decrease the energy barrier for *COOH formation, and guarantee the sufficient local *H near Pt surface. Additionally, the *H derived from H_2_O dissociation is favorable for the *CO hydrogenation pathway towards *CHO, eventually leading to the formation of CH_4_. This strategy might be easily applied to microenvironment and interface regulation in other electrocatalytic reactions.

## Teaser

The dominant hydrogen evolution activity on Pt surface is successfully converted to CO2 electroreduction activity via molecular modification, leading to the CH4 product in acid conditions.

## INTRODUCTION

Electrochemical reduction of CO_2_ presents a practical method for storing intermittent renewable energy in the form of chemical bonds [1,2]. This breakthrough allows for the closure of the carbon cycle, offering a sustainable solution for energy and environmental challenges. In the past, the focus on CO_2_ reduction has been principally on heterogeneous metal or metal oxide catalysts, most of them producing only two-electron products, CO or formate [3–5]. Among them, Cu-based materials are currently the most reported electrocatalysts capable of producing significant amounts of multi-electron products, e.g. CH_4_, CH_3_OH or C_2_H_4_ [6–8]. Nevertheless, stability of Cu-based materials still needs to be improved, especially in strongly acidic electrolytes. On the contrary, Pt-based catalysts are renowned for their remarkable stability in both acidic and alkaline electrolytes in various electrolytic reactions. However, Pt-based materials were still rarely utilized and studied in the CO_2_RR procedure, due to their strong and competing hydrogen evolution reaction (HER) activity in aqueous solution [9,10].

In comparison to a multitude of metallic catalysts, organic molecules present the benefit of exerting synthetic control over the steric and electronic characteristics in the proximity of the active sites [11]. Adding organic molecules into electrocatalytic systems can tune the CO_2_ interaction with the electrocatalysts for CO_2_RR [12], and enhance the adsorption of CO_2_ or stabilize the specific reaction intermediates [13]. Especially, additives of soluble organic promoters can act directly as catalysts, i.e. in combination with a noncatalytic electrode, or as cocatalysts to enhance the activity of the electrode [14]. As of now, tremendous efforts have been devoted to functionalize the surface of metal nanoparticles (NPs) with different organic ligands (e.g. small molecules, DNAs, proteins and polymers) [15–17]. Organic modification may introduce new degrees of freedom that regulate the properties of the metal interface, lower the overpotential, or even completely alter the reaction pathway. For example, it has been suggested that the chelating N-heterocyclic carbene (NHC) ligands bound to a Pd electrode can make an electron-rich Pd surface via the strong σ-donation from the NHC ligands to Pd, favoring the formation of formate product [18].

The traditional molecular-modified strategy involves physical adsorption or chemical reactions between the functional groups and the surface of NPs, depending on the composition of the NPs [19]. Especially, physical adsorption and Van der Waals forces are inherently unstable at the three-phase interface, where the electrocatalytic actions primarily take place. Consequently, types of composites called molecularly doped metals could be innovated to ensure the stable existence of organic modifiers at the interface. In these composites, metal crystallites act as a porous cage which stably confines specific ‘big’ modified molecules (e.g. nitrogen heterocycle) around the internal surface, yet allows the diffusion of ‘small’ molecules (e.g. CO_2_) [20–23]. This unique structure can regulate the properties of metal interfaces while generating the contact between reactants and active sites, which might be a promising methodology in CO_2_RR.

Herein, we utilized a molecular doping strategy to substantially entrap thionine (Th) molecules within Pt nanocrystalline and obtain a PtNPs@Th catalyst. These entrapped Th molecules are stably confined around the Pt surface and significantly alter the catalytic activity of the metallic Pt. Accordingly, the conventional HER activity on the Pt-based materials is suppressed to a great extent, but CO_2_RR performance is significantly enhanced in both strong acidic (pH 1) and weak acidic (pH 4.2) electrolytes. More importantly, the PtNPs@Th catalyst can maintain catalytic stability for more than 100 hours in acidic media, due to the strong corrosion resistance of metallic Pt. Combined with theoretical calculations, we further confirm that the synergistic effect of Th molecules and Pt contributes to the CO_2_RR towards CH_4_ production. This approach offers further opportunities to tailor the reaction interface with a molecular decorator in various electrocatalysis.

## RESULTS AND DISCUSSION

### CO_2_RR activity of Th molecules

**Figure 1.**
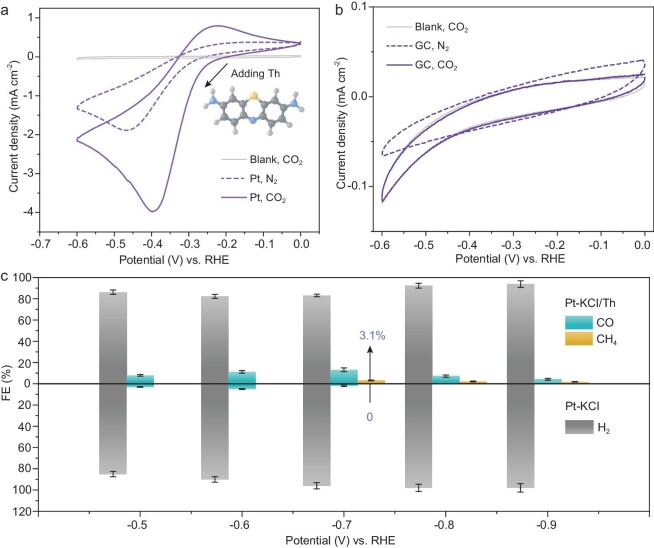
Electrochemical CO_2_ reduction tests recorded in 0.5 M KCl with 10 mM Th solution: (a) Pt electrode and (b) GC cathode; (c) FEs of all products using Pt cathode in CO_2_-saturated 0.5 M KCl solution dissolved with 10 mM Th.

The N-heterocyclic structure of Th molecules may induce CO_2_RR activity, which was first investigated using cyclic voltammetry (CV). The catalytic activity of Th for CO_2_ reduction was first explored by actual reaction. The CV tests were performed in N_2_- or CO_2_-saturated 0.5 M KCl aqueous solution with 10 mM Th using pure Pt, Pd and glassy carbon (GC) as the cathode. As shown in Fig. 1a, the introduction of Th triggered significant reduction peaks on the Pt cathode. In particular, saturation with CO_2_ led to a notably increased current density near −0.40 V vs. reversible hydrogen electrode (RHE) than that in N_2_-saturated solution, which might be connected to CO_2_ activation [24]. The Pd cathode can also achieve similar CO_2_ activation with the addition of Th, although the reduction peak shifts negatively to −0.53 V vs. RHE (Fig. S1). On the contrary, the GC electrode did not exhibit any distinct reduction peaks in N_2_- or CO_2_-saturated 0.5 M KCl solution, regardless of the addition of 10 mM Th (Fig. 1b). It might be attributed to the benzene ring or S element in Th molecules, which can interact with the metallic Pt or Pd surface and jointly acquire CO_2_RR activity [25,26].

As depicted in Fig. 1c, potentiostatic electrolysis was conducted to further verify the CO_2_RR performance of the Pt-KCl/Th molecule system, and the reduction products were detected via gas chromatography. Without the addition of Th in the KCl electrolyte, HER performance occupied an absolute dominant position on Pt electrodes. CO products from the CO_2_RR procedure can only be detected at lower potentials, and reach a maximum value of 4.9% faradaic efficiency (FE) at −0.6 V vs. RHE. After the addition of 10 mM Th into KCl electrolyte, although H_2_ is still the main product, the FE of CO is significantly improved to a maximum value of 13.1% at −0.7 V vs. RHE. Unexpectedly, CH_4_ product is also observed with a maximized 3.1% FE at −0.7 V vs. RHE. The Pt surface can generate a sufficient active hydrogen source, which might accelerate the production of CH_4_ (Fig. S2) [27]. The enhanced FE proves that Th modification does indeed have the potential to block HER and improve CO_2_RR performance on a Pt electrode. However, although plenty of Th molecules are dissolved in KCl electrolyte, it is difficult to ensure sufficient Th modifiers on the Pt surface during electrolysis (Fig. S3), due to the weak interaction and diffusion control kinetics (Fig. S4) [28]. Thereby, the inadequately modified Pt interface retains the main HER performance and restricts the FEs of CO and CH_4_.

### Synthesis and structural characterization of PtNPs@Th

**Figure 2.**
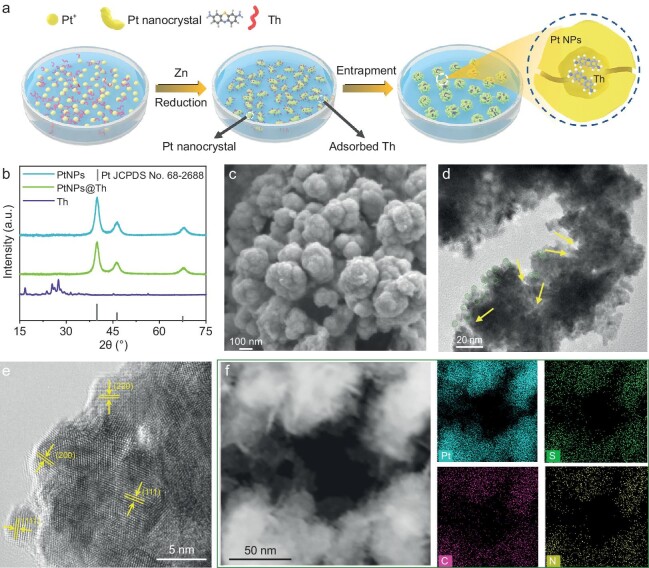
Synthesis and characterization images. (a) Schematic illustration of synthesis of PtNPs@Th. (b) XRD patterns of pure Th, pure PtNPs and PtNPs@Th. (c) The FE-SEM image of PtNPs@Th composite. (d) The TEM image of PtNPs@Th sample. (e) HR-TEM image of PtNPs@Th. (f) EDX elemental mapping images of PtNPs@Th, showing the signals of Pt, C, N and S.

A strategy of molecularly doped metals was employed to stably entrap abundant Th modifiers around the Pt interface. The entrapment of Th molecules in metallic Pt was realized through the reduction of Pt cations by Zn powder in a Th aqueous solution (Fig. 2a). Pt cations are first reduced into Pt nanocrystals and simultaneously adsorb Th molecules. The formation and aggregation of the Pt nanocrystals are faster than the adsorptive residence time of Th molecules, and then the Th molecules would be entrapped within Pt NPs, eventually obtaining PtNPs@Th (Fig. S5). If no Th was added in the reduction solution, pure Pt NPs would be obtained. The as-prepared PtNPs@Th and Pt NPs are both black powders displaying a certain metallic luster and good ductility. PtNPs and PtNPs@Th powders could be drop-casted onto conductive supports, or pressed into coins and directly used as cathodes in electrolysis (Figs S6, S7).

It is noteworthy that the entrapment is a fundamentally different process compared to adsorption, and is far more stable than pure adsorption [20,22]. If a PtNPs electrode was put in a Th solution and applied with a negative voltage, a portion of the Th molecules will adsorb on the PtNPs surface (denoted as PtNPs/Th), and this electrode was then removed and soaked in pure water. The resulting filtrate was analyzed using UV-vis spectra, which exhibited a characteristic absorption peak of Th at 600 nm (Fig. S8). As contrast, even if the PtNPs@Th was immersed in water for 24 hours, Th molecules will still not precipitate, indicating excellent stability in water. Intriguingly, the PtNPs@Th immersed in water was placed in a highly polar dimethyl sulfoxide (DMSO) solution, Th slowly separated out and exhibited a characteristic absorption peak in UV-vis spectra (Figs S9, S10 and Movie S1), revealing that Th was indeed entrapped into the Pt nanocrystals. To further confirm the content of Th molecules in PtNPs@Th, quantitative tests were conducted after the treatments of one-time water wash, two-time water wash or acid wash, as shown in Fig. S11. Interestingly, it was found that there was no considerable loss of Th during the preparation process evidenced by the relative concentration of filtrate. The encapsulation efficiency of PtNPs@Th was calculated to be ∼89%, indicating that the majority of Th was successfully encapsulated within the Pt nanocrystals rather than adsorbed on PtNPs surfaces.

Multiple technical methods were also utilized to characterize the as-prepared materials. The X-ray diffraction (XRD) pattern of the PtNPs@Th displayed the typical diffraction peaks of Pt (JCPDS No. 65–2868), while that of Th was not detected (Fig. 2b). Figure 2c and d show the field emission scanning electron microscopy (SEM) and transmission electron microscopy (TEM) images of PtNPs@Th, which has a uniform buds-like morphology. As shown in Fig. 2d, each small nanoparticle is composed of interconnected nanocrystals (green circles) surrounded with intercalated and marginal Th molecules (yellow arrows), indicating that Th may be encapsulated in PtNPs. The corresponding high-resolution TEM (HR-TEM) shows the specific lattice planes of (111), (200) and (220) of Pt (Fig. 2e). Moreover, EDX elemental mapping shows that C, N and S atoms are uniformly distributed across the bulk of the Pt, confirming the existence of Th molecules.

**Figure 3.**
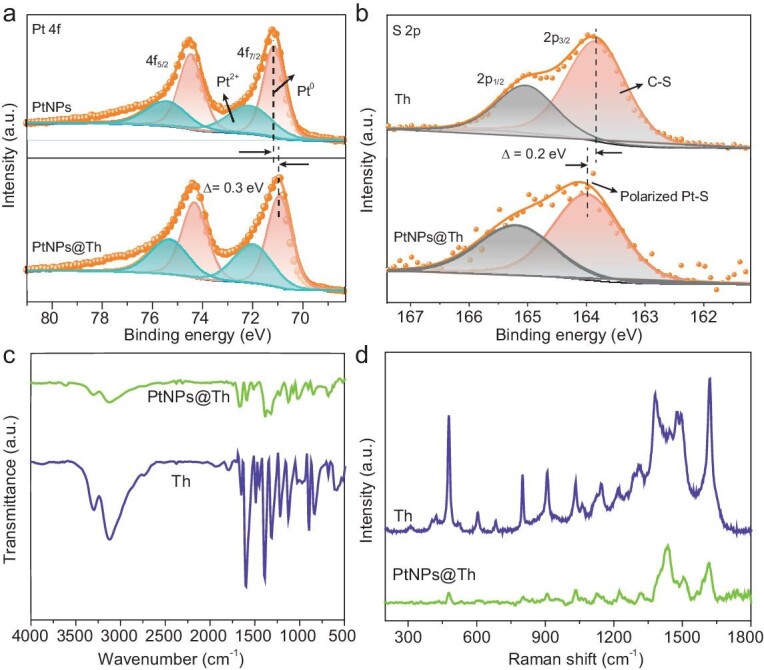
Structure characterizations of different catalysts. (a) High-resolution XPS spectra of Pt 4f for PtNPs and PtNPs@Th. (b) High-resolution XPS spectra of S 2p for pure Th and PtNPs@Th. (c) FT-IR spectra of pure Th and PtNPs@Th. (d) Raman spectra of pure Th and PtNPs@Th.

Furthermore, X-ray photoelectron spectroscopy (XPS), Fourier-transform infrared spectroscopy (FTIR), Raman spectroscopy and ultraviolet-visible (UV-vis) spectroscopy were employed to verify the occurrence of the immobilization of Th. The survey XPS spectrum of the PtNPs@Th indicates the presence of C, N, S and Pt elements (Fig. S12), suggesting that Th was connected to the surface of the platinum. The corresponding high-resolution XPS spectra of Pt 4f are displayed in Fig. 3a. Pt 4f XPS spectra exhibit two sets of peaks, corresponding to Pt^0^and Pt^2+^ [29,30]. Interestingly, the binding energy of Pt^0^ (4f_7/2_, 70.9 eV) for PtNPs@Th has a negative shift of 0.3 eV compared with that of pure Pt (4f_5/2_, 71.2 eV), suggesting the presence of strong interaction between the Th molecules and Pt. As shown in Fig. S13, the N 1 s spectra of PtNPs@Th could be deconvoluted into two species, –N= (398.4 eV), C–NH_2_ (399.7 eV), respectively, the same as that in Th molecules [31]. The S element in Th with S 2p_3/2_ and 2p_1/2_ binding energies are 163.8 eV and 165.0 eV, respectively. In comparison, the S element in PtNPs@Th exhibits the S 2p_3/2_ binding energy of 164.0 eV and S 2p_1/2_ binding energy of 165.2 eV (Fig. 3b). These findings suggest that there is a charge transfer from S to Pt, forming a polarized Pt–S bond [32]. FTIR spectra of Th and PtNPs@Th are shown in Fig. 3c. Th exhibits distinct peaks in the frequency range of 500∼1750 cm^−1^. Similarly, the PtNPs@Th sample also demonstrates those characteristic absorbance peaks at 854 and 901 cm^−1^ due to the C–S bending vibration of Th, and 1383, 1480 and 1587 cm^−1^ ascribed to ring vibration of Th [31], suggesting that Th was connected to the Pt surface. Meanwhile, in the Raman spectra of PtNPs@Th, the same peaks at 479, 909, 1032, 1382, 1480 and 1620 cm^−1^ correspond to the spectrum of Th (Fig. 3d) [33], which further confirm the entrapment of Th into Pt nano-cages. The above results demonstrate that Pt and Th retain respective physical and chemical properties, accompanied with electronic interaction between them.

### CO_2_RR performance of PtNPs@Th in acidic media

The CO_2_RR activity of PtNPs@Th was evaluated by linear sweep voltammetry (LSV) studies in CO_2_-saturated 0.5 M KCl (Fig. S14a). The PtNPs@Th catalyst exhibits larger total current densities and positively shifts in onset potentials relative to PtNPs catalyst in CO_2_ atmosphere. The onset potential of PtNPs@Th catalyst is only about −0.3 V vs. RHE, and an obvious reduction peak appeared at −0.6 V vs. RHE. Under N_2_ atmosphere, the PtNPs@Th catalyst has less total current density and negatively shifts onset potentials relative to PtNPs catalyst (Fig. S14b). This indicates that the PtNPs@Th catalyst could inhibit HER. Therefore, Th might play a vital role in the high performance CO_2_RR. The double-layer capacitance (*C*_dl_) was used to estimate the electrochemical active surface area (ECSA). It is shown that the PtNPs@Th electrode has larger *C*_dl_ than PtNPs electrode (Fig. S15). In addition, the underpotential deposition of hydrogen experiment was also performed to calculate the ECSA of Pt-based materials. As shown in Fig. S16, the ECSA of the PtNPs@Th was 20 m^2^ g_Pt_^−1^, which was greater than that of the PtNPs (13 m^2^ g_Pt_^−1^), favorable to the improvement of CO_2_RR activity. The outstanding performance of the PtNPs@Th electrode, in comparison to the PtNPs electrode, can be attributed to the modified microenvironment created by the addition of Th. The kinetic processes were further investigated using electrochemical impedance spectroscopy (EIS). PtNPs@Th displays a smaller Nyquist semicircle diameter compared to that of PtNPs (Fig. S17), suggesting the fast charge-transfer kinetics in CO_2_RR.

**Figure 4.**
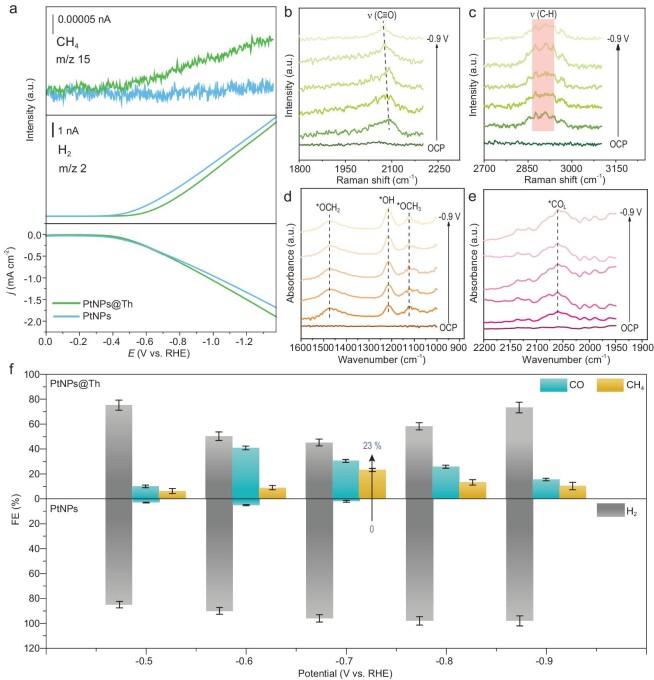
*In-situ* characterizations and FEs of reduction products using different catalysts for the CO_2_RR procedure. (a) LSV curves at PtNPs@Th with the corresponding mass fragment signals of online DEMS. (b, c) *In-situ* Raman spectra and (d, e) *in-situ* FTIR spectra on PtNPs@Th electrode as a function of the applied cathodic potential. (f) Faradaic efficiency of all products at PtNPs@Th and Pt NPs.

Furthermore, to gain the underlying electrochemical behaviors of the catalysts under a real electrolysis process, online electrochemical mass spectrum (DEMS) measurements were performed. It can be found in Fig. 4a that the mass-to-charge ratio (m/z) signals at 2 and 15 assigning to H_2_ and CH_4_, respectively, start to emerge at −0.4 V on PtNPs@Th catalyst. In contrast, the PtNPs sample has no CH_4_ signal in the whole potential range, suggesting that entrapping Th molecules into Pt nano-cages would lead to an increase of the selectivity of CH_4_. Moreover, *in-situ* Raman spectroscopy was executed in order to observe the intermediates during the CO_2_RR (Fig. 4b and c). In detail, the peaks around 2070 cm^−1^ are assigned to the C≡O stretching vibrations, *CO species have been demonstrated to be the key intermediates towards CH_4_ and CO production. In addition, the obvious Raman bands located at 2800–3000 cm^−1^ appeared at −0.5 V vs. RHE, which was attributed to the C–H stretching vibration [34,35]. More impressively, *in-situ* FTIR spectroscopy depicted in Fig. 4d and e reveal that two peaks are centered at 1475 cm^−1^ and 1121 cm^−1^, attributing to *OCH_2_ and *OCH_3_, respectively, which are the key intermediates for CO_2_RR to CH_4_ [36,37], but they are not detected for PtNPs (Fig. S18). In addition, the peak at ∼2060 cm^−1^ assigning to the stretching modes of linear-adsorbed *CO (*CO_L_) is observed for the PtNPs@Th sample in a wider voltage range of *CO coverage compared with that of PtNPs [38]. The strong *CO adsorption facilitates its hydrogenation on PtNPs@Th to the *OCH_2_ intermediate and further to the *OCH_3_ intermediate via a series of proton-coupled electron transfer reactions [39]. The above results suggest that entrapping Th molecules into Pt nanocrystals would lead to an increase of the selectivity of CH_4_.

Based on the quantitative analysis of gas chromatography, the reduction products of using PtNPs and PtNPs@Th electrodes are fundamentally different, as shown in Fig. 4f. For PtNPs@Th, the highest FE of CH_4_ (23.0%) was achieved at −0.7 V vs. RHE, accompanied by a certain amount of CO (9.8% to 30.2% FE). On the contrary, PtNPs produces H_2_ with >90% FEs and almost negligible CH_4_, and only a trace amount of CO could be detected with 2.1% to 4.8% FEs. In addition, PtNPs@Th generates much larger partial current densities of CO and CH_4_ than those of PtNPs (Figs S19–S21). The CO_2_RR performances of PtNPs with different Th loading amounts were also investigated (Fig. S22 and Table S1). It could be found that the content of Th has an apparent impact on the product distribution. The encapsulation of Th in PtNPs could inhibit the process of H_2_ evolution; FE_CO_ and FE_CH4_ have a positive relation with the encapsulation amount of Th. The above results further demonstrate that the entrapped Th molecules can suppress the excessive H_2_ evolution of metallic Pt. In addition, the CO_2_RR performances of PtNPs@Th and PtNPs were assessed in a flow cell device with a gas diffusion electrode. The maximum FE of CH_4_ is 20.8% at −0.9 vs. RHE, which does not increase significantly compared with those in the conventional H-type cell (Fig. S23). Besides, it can be observed from the ^13^CO_2_ isotope experiment that the C element in CH_4_ product does indeed come from the reduction of CO_2_, rather than decomposition of organic modified molecules (Fig. S24). These results are consistent with those of online DEMS, *in-situ* Raman and FTIR spectra, indicating that the modification of the Th molecules totally change the dominant HER activity to CO_2_RR activity on the Pt surface.

As mentioned above, Cu-based materials can produce considerable hydrocarbons, but their stability is poor in acidic media. In contrast, PtNPs@Th catalyst could still maintain CO_2_RR activity (Fig. S25), and produce CH_4_ with a 19.1% FE and CO with a 25.2% FE (Fig. S26) in strong acid electrolyte (pH 1), which is also comparable to other published works (Table S2). Besides, PtNPs@Th was subjected to a long-time electrolysis at the potential of −0.7 V vs. RHE. No significant decrease in current density and FEs was observed during 100 hour tests (Figs S27, S28) and the pH values stabilized in strong acid electrolyte near 1 to 1.15 (Fig. S29), suggesting that the electrolyte environment was relative stable when electrolyzed under moderate potential range. The structural stability of the sample was confirmed by a series of comparative characterizations for the electrodes after CO_2_RR test. The electrolyte after the reaction was tested using UV-vis spectroscopy, which demonstrated that Th did not dissociate into the electrolyte during long-term electrolysis (Fig. S30 and Movie S2). The EDX elemental mapping of the sample result reflects that Th was steadily encapsulated by PtNPs during electrocatalysis (Fig. S31). The N and S electronic states showed no obvious change after CO_2_RR as proven by XPS data (Fig. S32). FTIR and Raman spectra after CO_2_RR also support that the PtNPs@Th sample has good stability (Figs S33, S34). This stable CO_2_RR performance is ascribed to the *in-situ* immobilization through covalent bonds as it effectively inhibits the dissolution of Th in the electrolyte.

### Theoretical research on PtNPs@Th

**Figure 5.**
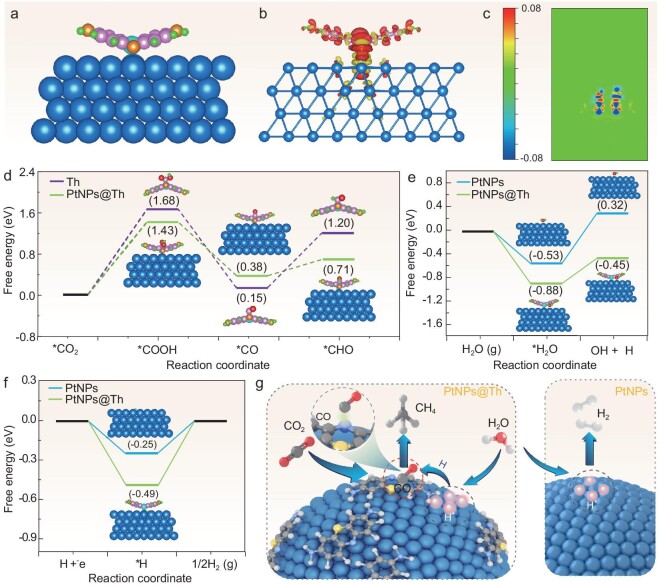
DFT calculated free energy diagram for CO_2_RR procedure. (a) Side view of Th-Pt (111) model structure for DFT calculations. Blue: Pt atom, cyan: S atom, orange: N atom, green: H atom, pink: C atom. (b, c) Charge density differences of Th-Pt (111). (d) The calculated Gibbs free energy on Th and Th-Pt (111). (e) Calculated energy barriers of water dissociation on Pt and Th-Pt (111) surfaces. (f) Gibbs free energy of *H adsorption for Pt (111) and Th-Pt (111). (g) Simplified schematic representation of CO_2_ electrochemical reduction to CH_4_ on PtNPs@Th catalyst.

We further carried out density functional theory (DFT) calculations to unravel the electronic properties as well as the CO_2_RR mechanism of PtNPs@Th catalyst. The micro-environment exhibited by the PtNPs@Th structure on the electrode surface is responsible for the high selectivity when obtaining CH_4_. Taking this as the premise, Pt (111)-Th structures for PtNPs@Th are modeled and examined (Fig. 5a and Fig. S35), and the differential charge density was calculated (Fig. 5b and d). It can be found that significant electron transfer occurred at the Pt and Th interface, resulting in reduced electron density around the S atom. In addition, the charge difference map of PtNPs@Th also shows an accumulation of charge in the region between Pt and S atoms, which is consistent with the XPS result for the formation of Pt–S bonds. To gain a deeper insight into the CH_4_ formation mechanism on PtNPs@Th, we systematically investigated the process of CO_2_RR on its surface. The adsorbed intermediate structures are shown in Figs S36–S44. According to the experimental result, the N in nitrogen-heterocycle in Th was manipulated into acting as the reactive site for CO_2_ reduction and Pt substrate supplying the H availability. As shown in Fig. 5d, the *CO_2_ and *CO hydrogenation energy on the pure Th surface are 1.68 and 1.05 eV, respectively, which is apparently higher than that on Th-Pt (111) surface (1.43 eV and 0.33 eV) and lower than on Pt (111) surface (2.83 eV and 0.92 eV, Fig. S45), respectively, suggesting that the *CO hydrogenation process occurs more easily on N site than Pt site. It is reasonable to infer that the existence of Pt is favorable for the hydrogenation processes. However, there is insufficient local *H availability for the pure Th in CO_2_RR. Furthermore, the water dissociation and hydrogen evolution processes were calculated to analyze the *H availability (Figs S46–S51). Specifically, as depicted in Fig. 5e, the Th-Pt surface has a lower water adsorption energy (−0.88 eV vs. −0.53 eV) and a lower dissociation barrier (0.43 eV vs. 0.85 eV) compared with Pt (111), indicating that upon anchoring Th molecules, it facilitates the H_2_O adsorption and dissociation, and produce surface protons. Furthermore, it can be found that the free energy of *H on Th-Pt (111) surface is −0.49 eV, lower than that of pure Pt (−0.25 eV), indicating the strong *H binding energy, which prevents them from donating *H for quick H_2_ evolution process (Fig. 5f). The above results show that the existence of Th helps stabilize proton adsorption and guarantee the sufficient local protons near Pt, synergistic catalyzing *CO hydrogenation to CH_4_.

There have been numerous reports on the reaction mechanism of nitrogen-heterocycle catalytic systems [40]. Studies using nitrogen-heterocycle carbene-loaded Au electrodes [4], homogeneous pyrolysis [41] and its derivatives [24] have proposed speculated mechanisms, highlighting the significant role of N active sites on the nitrogen-heterocycle in the CO_2_RR process. Intrigued by theoretical calculations and experimental measurements, the catalytic mechanism of PtNPs@Th can be inferred, as summarized in Fig. 5g. For PtNPs@Th, the N in the nitrogen-heterocycle as the active sites chemically adsorb and activate CO molecules, while Pt supplies sufficient electrons and *H intermediates to facilitate one-electron, one-proton transfer, resulting in the formation of *COOH intermediates. Subsequently, the *COOH intermediates gain an additional electron and proton, leading to the production of *CO. During this step, a majority of the CO molecules desorb from the catalyst and form products, while some *CO is adsorbed by the N catalytic site and undergoes subsequent 6-electron reactions. Th on the surface of Pt inhibits the hydrogen evolution of Pt. At the same time, the metal conductivity of Pt provides electrons for Th reduction of CO_2_, which leads to the formation of CH_4_. These factors make Pt more suitable for generating intermediates. In other words, the N active sites, which adsorb stable *CO intermediates, are surrounded by the abundant amounts of *H, and Pt contributes a large number of electrons, enabling rapid coupling of these two intermediate products to produce CH_4_.

## CONCLUSION

In summary, we prepared a heterogeneous PtNPs@Th composite by the entrapment of abundant Th molecules within Pt nanoparticles. Pure Pt produces H_2_ with >90% FEs and only a trace amount of CO. After the modification of Th molecules, HER activity is greatly suppressed on PtNPs@Th composite, and the FEs of CO was significantly enhanced in acidic media. Intriguingly, CH_4_ with ∼20% FEs was also detected using Pt-based catalysts in CO_2_RR for the first time. Theoretical calculations disclose that the existence of Th guarantees the sufficient local proton near the Pt surface, synergistically catalyzing *CO hydrogenation to CH_4_. Furthermore, PtNPs@Th composite has excellent 100-hour stability and reusability in strong acidic electrolyte (pH 1), which is obviously superior to traditional Cu-based electrocatalysts. This research highlights the importance of modified interface and microenvironment in the preparation of hybrid materials, and also provides a promising strategy for the fabrication of high-performance electrocatalysts in acidic media.

## METHODS

### Synthesis of PtNPs@Th catalyst

A certain amount of K_2_PtCl_4_ and sodium lauryl sulfate (mass ratio: 20/1) was dissolved in 25 mL of distilled water, and was poured into a stirred solution of Th (0.06 mmol) in 25 mL of water. After stirring, zinc powder was added, and the black slurry was stirred for another 6 hours. The precipitate was filtered, washed with water and dried overnight. Dark powder was achieved, then pressed into a coin and directly used as the cathode. Pure Pt NPs were prepared in the same way as PtNPs@Th except for the absence of Th in the reducing solution. If PtNPs was put in a Th solution and applied with a negative voltage, a portion of the Th molecules will adsorb on the PtNPs surface, which can be denoted as PtNPs/Th.

### Electrolysis, products analysis and quantification

An electrochemical workstation (CHI 760E, Shanghai CH instruments Co., China) was used for electrochemical reaction experiments. PtNPs@Th powder was compacted into a coin and used as the cathode to test the catalytic activity for CO_2_ electroreduction in a conventional H-type cell with 0.5 M KCl as the electrolyte. Before each set of the test, the electrolyte was bubbled with CO_2_ for 30 min until a CO_2_-saturated solution was reached. Another electrolyte was composed of 0.1 M HCl and 0.5 M KCl (pH 1) aqueous solution.

Briefly, the gaseous products were analyzed by gas chromatograph with a flame ionization detector (FID) and a thermal conductivity detector (TCD). We also analyzed the liquid products by ^1^H-NMR with a water suppression method. Cyclic voltammetry was conducted in 0.5 M KCl at a scan rate of 50 mV s^‒1^, and ECSAs were estimated using the hydrogen adsorption (H_UPD_) charge determined by excluding the hydrogen evolution area and the Coulombic charge required for oxidation on polycrystalline Pt (210 μC cm^−2^).

## Supplementary Material

nwae361_Supplemental_Files
