# Supplementary material for: Molecular modification enables CO2 electroreduction to methane on platinum surface in acidic media
Source: Natl Sci Rev. 2024 Nov 19;11(12):nwae361. doi: 10.1093/nsr/nwae361 (PMC11631074; doi:10.1093/nsr/nwae361)
Supplement: nwae361_Supplemental_Files [file nwae361_supplemental_files.zip › 819-Supporting information.pdf]

Supplementary information

Molecular Modification Enables CO_2 Electroreduction to Methane on Platinum Surface in Acidic Media

Hengpan Yang,† Huizhu Cai,† Deliang Li, Yan Kong, Shangzhao Feng, Xingxing Jiang, Qi Hu, and Chuanxin He*

College of Chemistry and Environmental Engineering, Shenzhen University, Shenzhen, Guangdong, 518060, China.

†These authors have equal contributions.

*Corresponding authors.

E-mail addresses: hecx@szu.edu.cn (C. He)

Contents:

Supplementary Figures S1 to S51

Supplementary Tables S1 to S2

Supplementary Movies S1 to S2

Methods

Structural analysis

SEM were analyzed using Hitachi S-4800 field emission Scanning Electron Microscope (FE-SEM). Transmission electron microscopy (TEM) images was analyzed by JEM-2100F field emission electron microscope conducted at an accelerating voltage of 120 kV. X-ray diffraction (XRD) patterns were recorded by an Ultima IV X-ray powder diffractometer using Cu Kα radiation (k= 1.5406 Å). X-ray photoelectron spectra analysis (XPS) was recorded with ThermoVG Scientific ESCALAB 250 X-ray photoelectron spectrometer (Thermo Electron, U.K.) using Al Kα X-ray source. The dopant amount in filtrate were determined by a high-performance liquid chromatography (HPLC) instrument (DIONEX Ultimate 3000 pump) equipped with a UV (RS Variable Wavelength) detector. Infrared spectra were achieved by a Fourier transform infrared spectrometer (FTIR, SHIMADZU). UV spectrum is achieved by Ultraviolet-visible spectrophotometer (UV-vis, SHIMADZU).

In-situ Raman spectroscopy was carried out with a Renishaw inVia Raman spectrometer in a homemade gear and a 50× water immersion lens. The CO_2 electrocatalytic reduction on PtNPs@Th electrodes were performed in CO_2-saturated $KHCO_3$ solution with a flow pump to fresh the reaction electrolyte. Meanwhile, the in-situ Raman spectroscopy was applied to probe the reaction process, and the wavelength of the excitation laser was 532 nm. The acquisition time is 60 s for each spectrum with the accumulation times of 2.

In-situ differential electrochemical mass spectrometry (DEMS) measurements were used to detect intermediates and products of the CO_2RR process. CO_2-saturated 0.5 M KCl as an electrolyte. The voltage range during the test was 0 to −1.3 V vs. RHE at a scan rate of 5 mV s^{-1}. After the baseline was kept steady, the possible intermediate and product were collected during the electrocatalysis process. The signal of mass-to-charge ratio (m/z) of 2 and 15 represented the products of H_2 and CH_4, respectively. The isotope labeling experiment was conducted using $^{13}CO_2$ atmosphere.

In-situ FTIR spectra was carried out with Bruker IINVENIO-S. Testing was conducted in a homemade in-situ cell, with Ag/AgCl used as a reference. The reference electrode was introduced near the working electrode, and a Pt wire served as the counter electrode. All spectra were presented as $\Delta R/R=(Es-E_R)/E_R$, where Es and E_R represent the sample and background spectra, respectively. The spectral resolution was set at 4 cm^{-1} for all measurements unless otherwise specified.

Computational details

$*CO_2$ to *CHO reaction pathway for thionine and PtNPs@Th, water dissociation and hydrogen evolution processes were studied by density functional theory (DFT). All calculations were performed via Vienna ab initio simulation package 6.1.0 (VASP)[1] with the projector augmented wave (PAW) pseudopotentials.[2] The exchange-correlation interaction was calculated by the generalized gradient approximation (GGA) method with the Perdew-Burke-Ernzerhof (PBE) pure functional.[3] The long-range van der Waals (vdW) interactions were handled by Grimme's DFT-D3 scheme.[4] Cubic platinum cell (fcc) was constructed based on XRD measurements. Afterwards, the corresponding thionine molecular was placed on the Pt (111) slab to model the composite catalyst. For the parameters of calculations, the kinetic energy cutoff for the plane-wave basis set was chosen as 450 eV. The electronic energy was considered self-consistent when the energy change was smaller than 10^{-5} eV. Gaussian smearing with a width of σ=0.1 eV was set for the occupation of the electronic levels.[5] As for the structure optimization, all atoms were allowed to fully relax with the conjugate gradient method until the maximum force on any atom was smaller than 0.02 eV/Å. The Brillouin zone was sampled with 2×2×1 Monkhorst-Pack k-points grids.

Vibrational frequency calculations confirmed that the optimized structures have no imaginary frequency, i.e., they locate at the minima of potential energy surface, and the Gibbs free energy is expressed as

$$\Delta G=\Delta E-T\Delta S+\Delta ZPE \qquad (1)$$

where ΔE is DFT-calculated reaction energy in vacuum, TΔS is the entropy contributions to the reaction at T=298.15 K, ΔZPE is zero-point energy (ZPE) correction based on the calculated vibrational frequencies. It should be noted that the reaction pathways were calculated from the computational hydrogen electrode (CHE) model according to the study of Nørskov et al.[6]

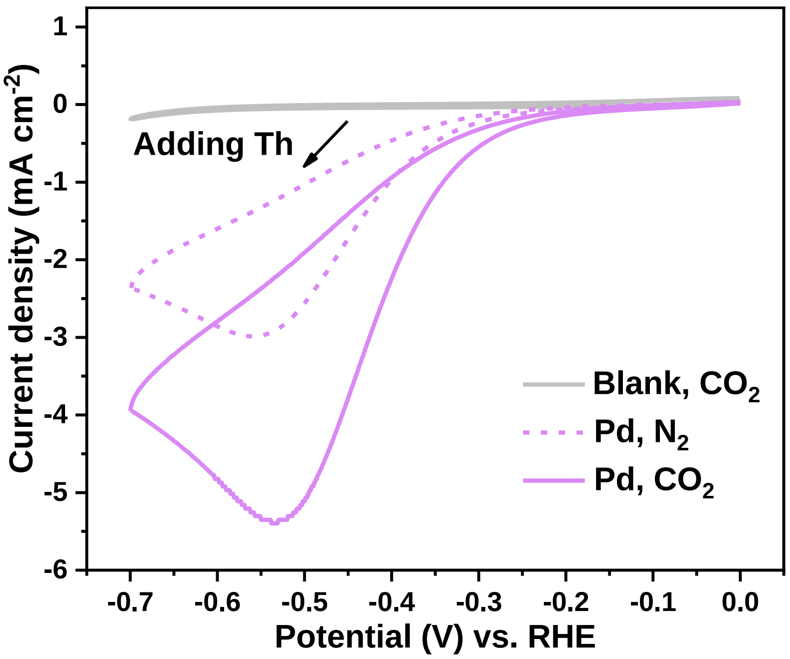

Figure S1. Cyclic voltammograms recorded at a sweep rate of 0.1 V s^{-1} in 0.5 M KCl dissolved with10 mM Th solution at Pd cathode.

As depicted in Figure S1, an irreversible reduction wave was observed at −0.53 V vs. RHE on the Pd electrode in the N_2 saturated solution, whereas in the CO_2 saturated solution, the current density of the Pd electrode was amplified and the potential slightly shifted toward positive values. Hydrogen is known to be bound more weaker to other metals, like Pd, than Pt, the reduction process was observed at more negative potentials on Pd electrodes.

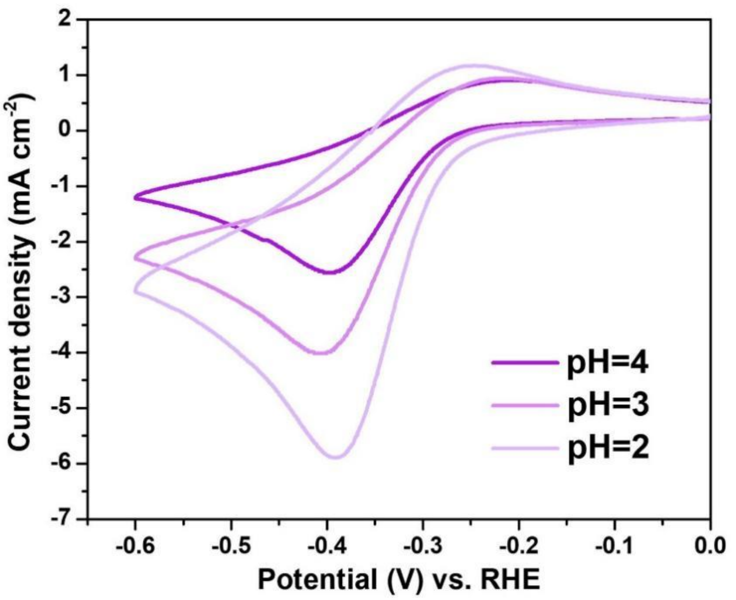

Figure S2. Cyclic voltammograms recorded at a sweep rate of 0.1 V s^{-1} in 0.5 M KCl dissolved with 10 mM Th solution at Pt cathode saturated of N_2 with different pH values of 4, 3 and 2.

It is evident that Th displays a reversible reduction mechanism in the presence of CO_2 at pH 3. The reduction current significantly rises as the pH decreases to 2. Conversely, when the pH value is increased to 4, the reduction current drops to 2/3 of that observed at pH 3. This observation indicates the critical role of proton in thionine catalytic system.[7]

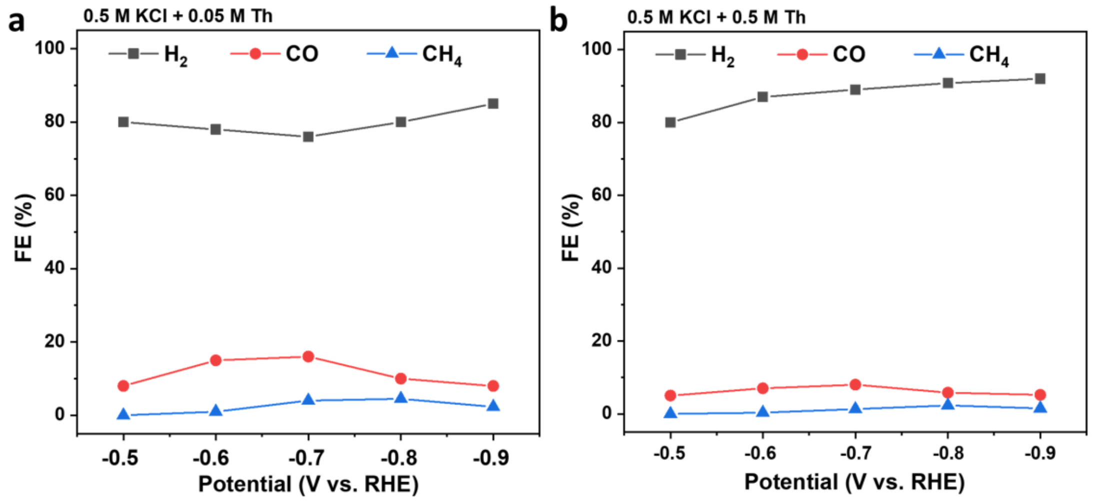

Figure S3. FEs of all products using Pt electrode in CO_2-saturated 0.5 M KCl solution dissolved with 0.05 M (a) and 0.5 M (b) Th.

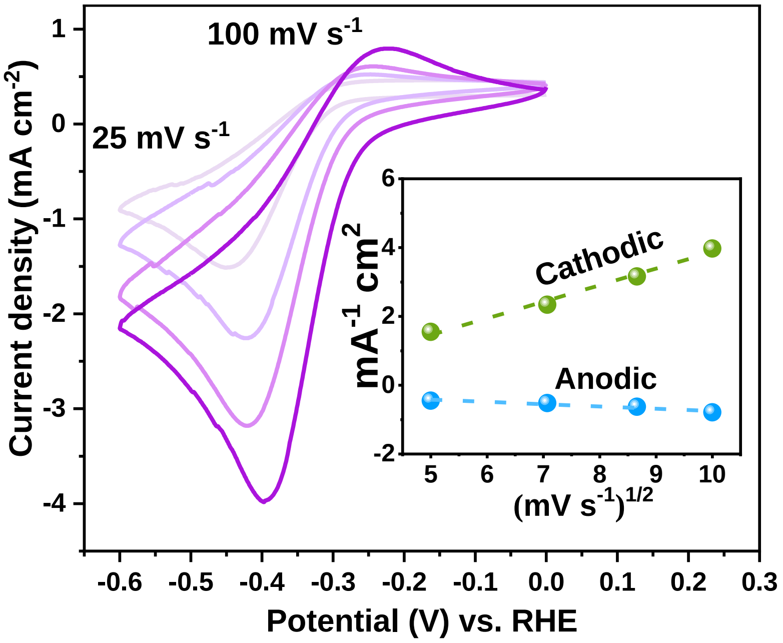

Figure S4. Cyclic voltammograms recorded at different rates in 0.5 M KCl and 10 mM Th solution saturated of CO_2 at Pt cathode.

Figure S4 presented a correlation between peak anode and cathode current densities, demonstrating a linear relationship with the square root of the scanning rate. This suggests the presence of a diffusion-limited electrochemical reaction, in line with the Randles-Sevcik equation.[8]

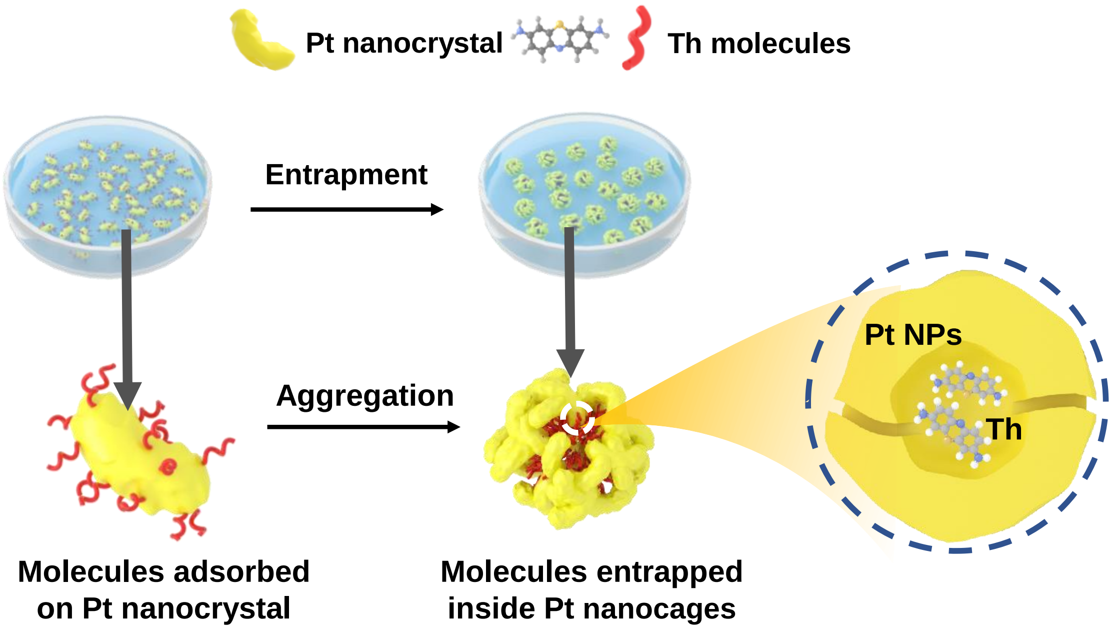

Figure S5. Scheme of aggregation and entrapment mechanism.

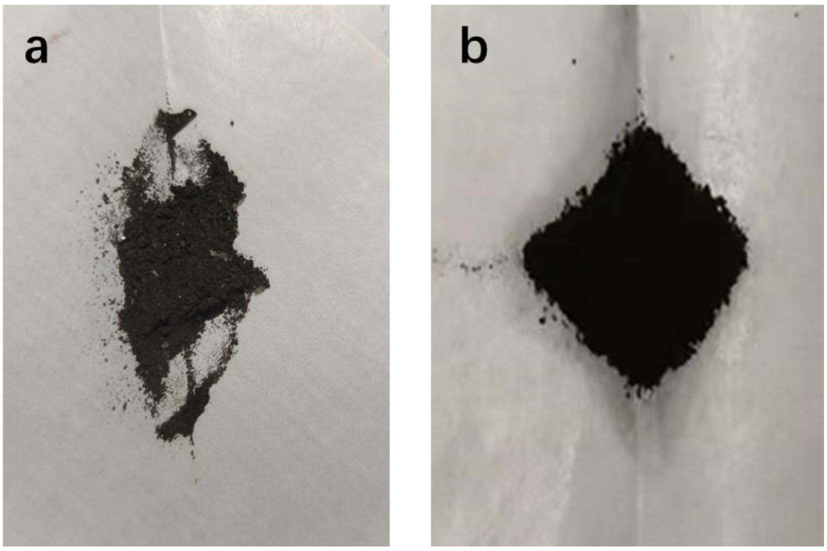

Figure S6. Pure PtNPs (a) and PtNPs@Th (b), both of which are black powder with a metallic luster.

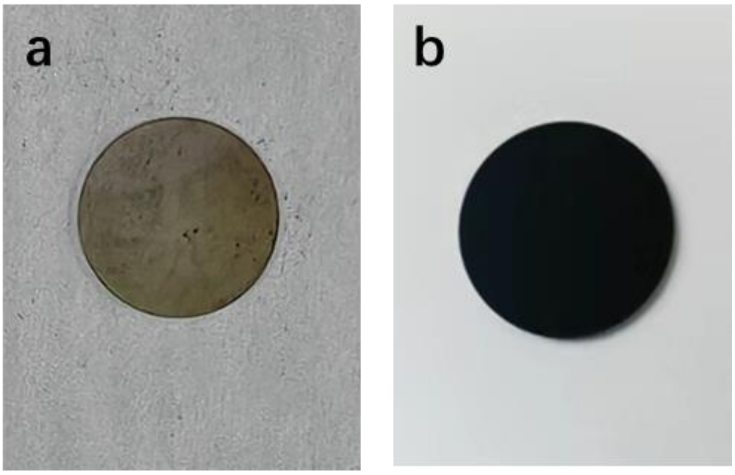

Figure S7. Pure pressed PtNPs coin (a) and pressed PtNPs@Th coin (b), both of which maintain good malleability.

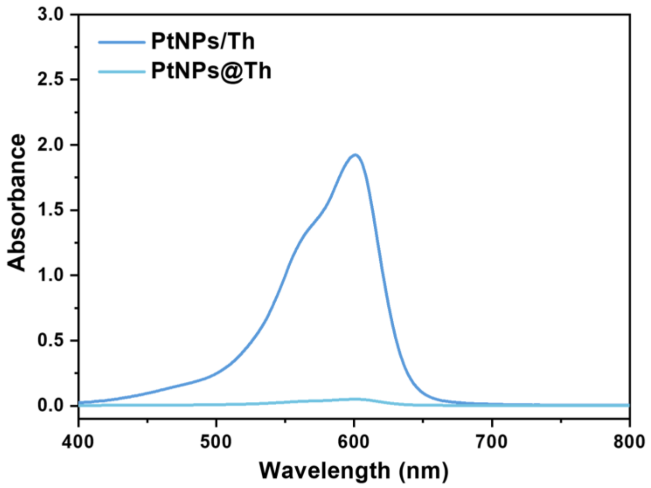

Figure S8. UV-vis spectrogram of the PtNPs/Th and PtNPs@Th solution after soaking in water.

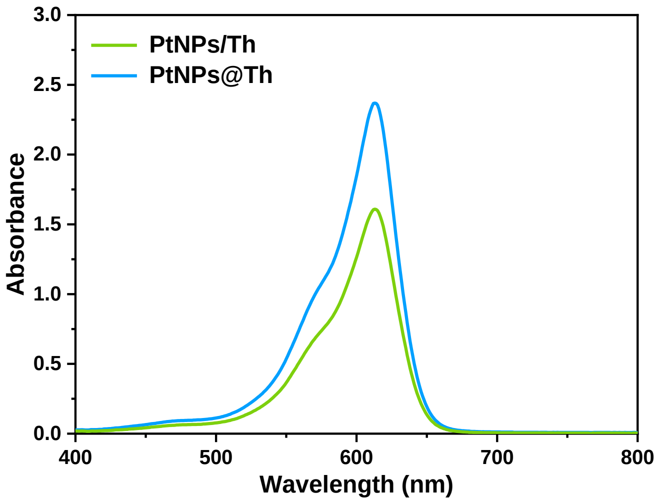

Figure S9. UV-vis spectrogram of the DMSO extracted PtNPs/Th and PtNPs@Th solution.

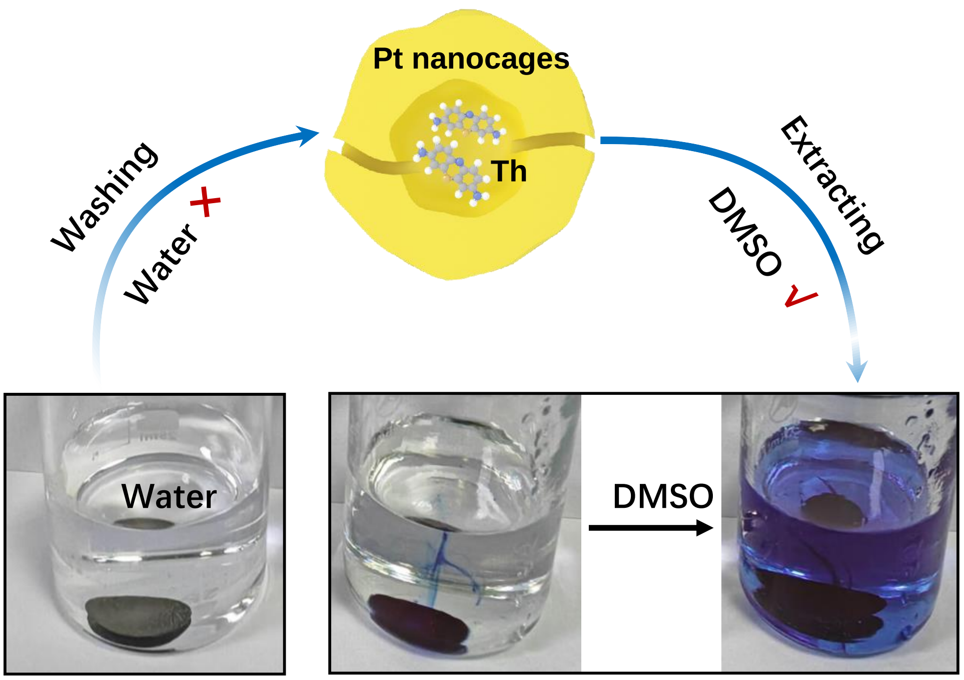

Figure S10. PtNPs@Th in water (left) and DMSO (right); PtNPs@Th is stable in aqueous electrolyte, but Th molecules within PtNPs@Th can be extracted out using strong polar solvents, such as DMSO.

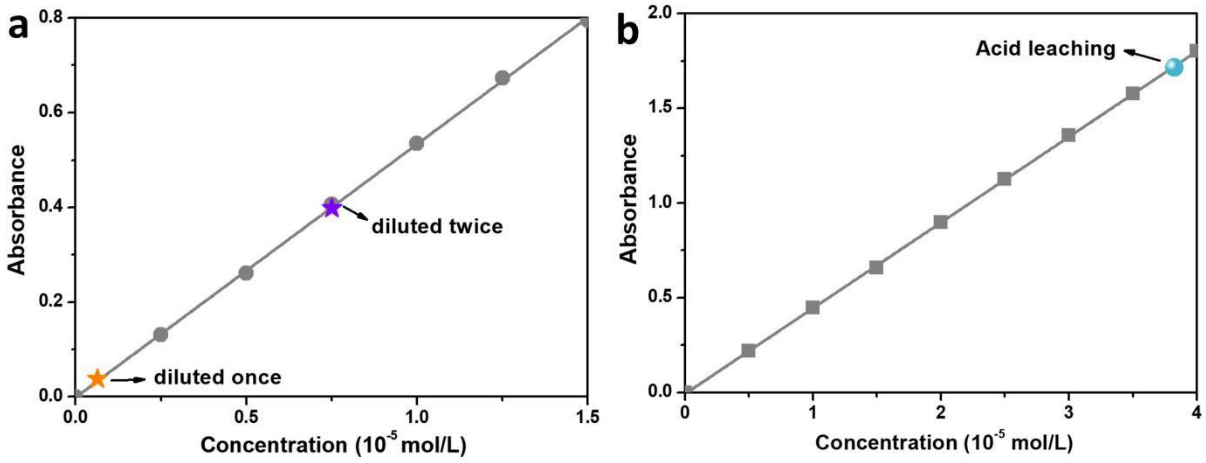

Figure S11. The ultraviolet spectrum test of PtNPs@Th filtrate, the water filtrate (a) and the hydrochloric acid filtrate (b).

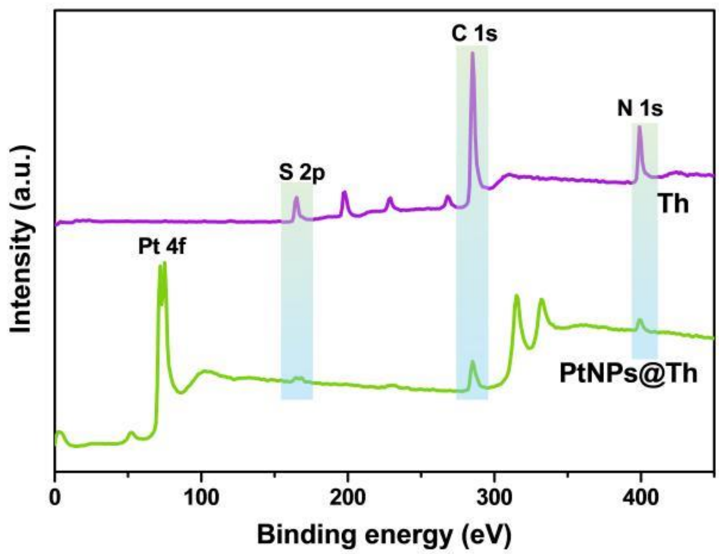

Figure S12. XPS survey spectra of Th and PtNPs@Th.

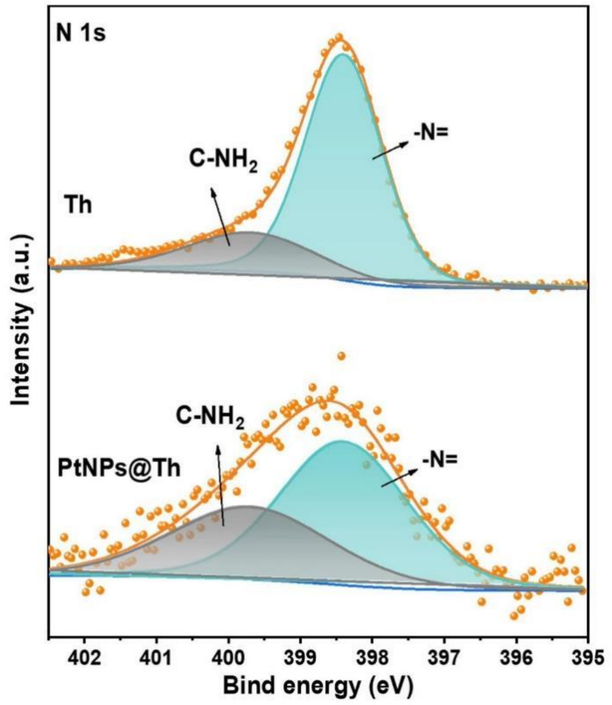

Figure S13. High-resolution XPS spectra of N 1s for Th and PtNPs@Th.

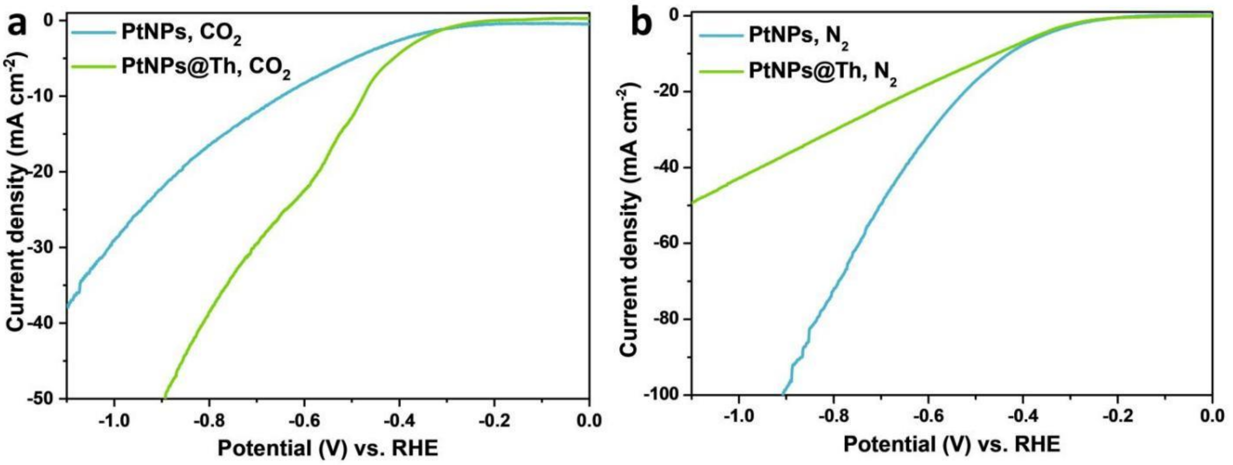

Figure S14. (a) LSV curves of PtNPs and PtNPs@Th coins under CO_2-saturated 0.5 M KCl. (b) LSV scans of PtNPs and PtNPs@Th coins under N_2-saturated 0.5 M KCl.

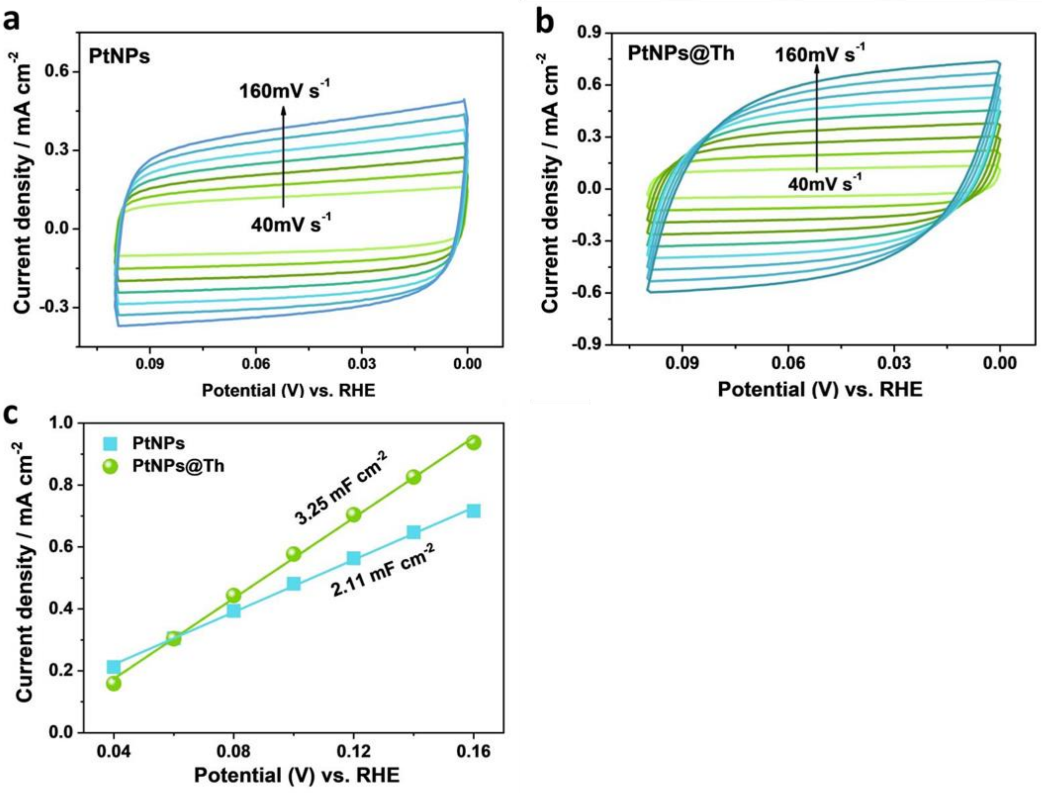

Figure S15. CV curves at different scan rates for PtNPs (a), PtNPs@Th (b), (c) Scan rate dependence of the current densities of PtNPs and PtNPs@Th.

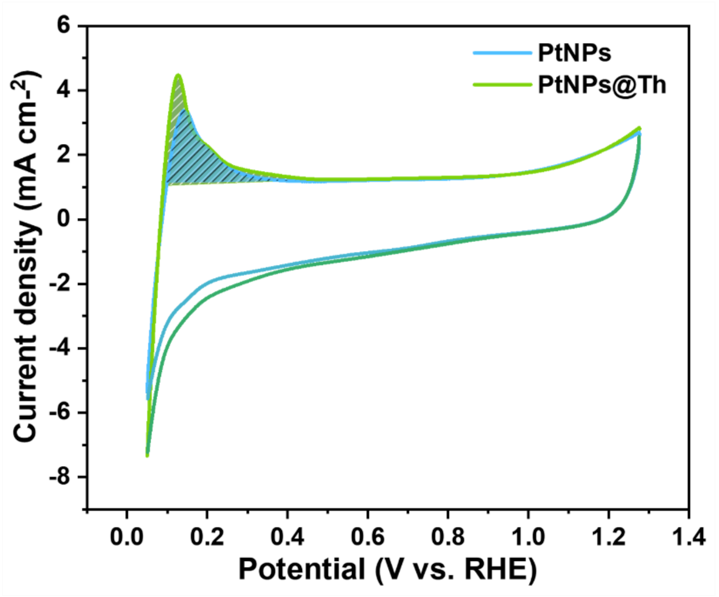

Figure S16. Cyclic voltammogram of PtNPs and PtNPs@Th collected in 0.5 M KCl at a scan rate of 50 mV s^{-1}.

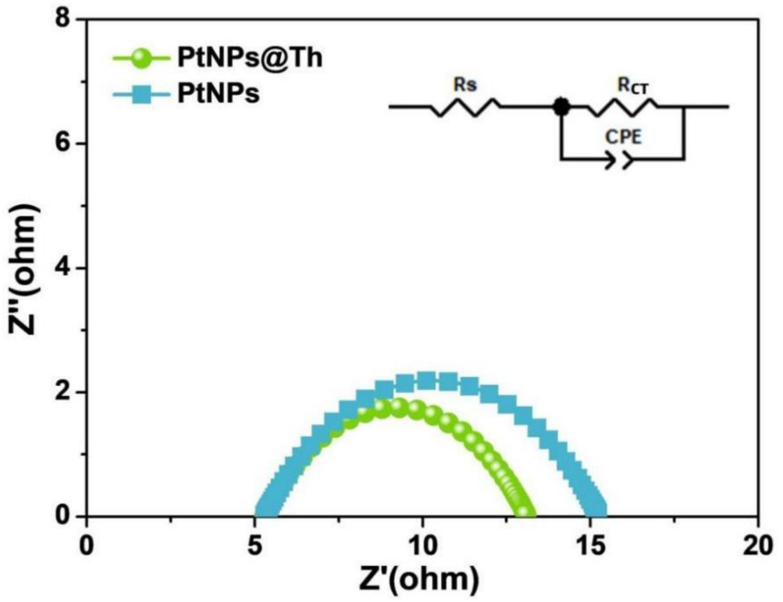

Figure S17. Electrochemical impedance spectroscopy (the inset is the fitting equivalent circuit that corresponds to the Nyquist plots) of PtNPs and PtNPs@Th tested in 0.5 M KCl saturated with CO_2.

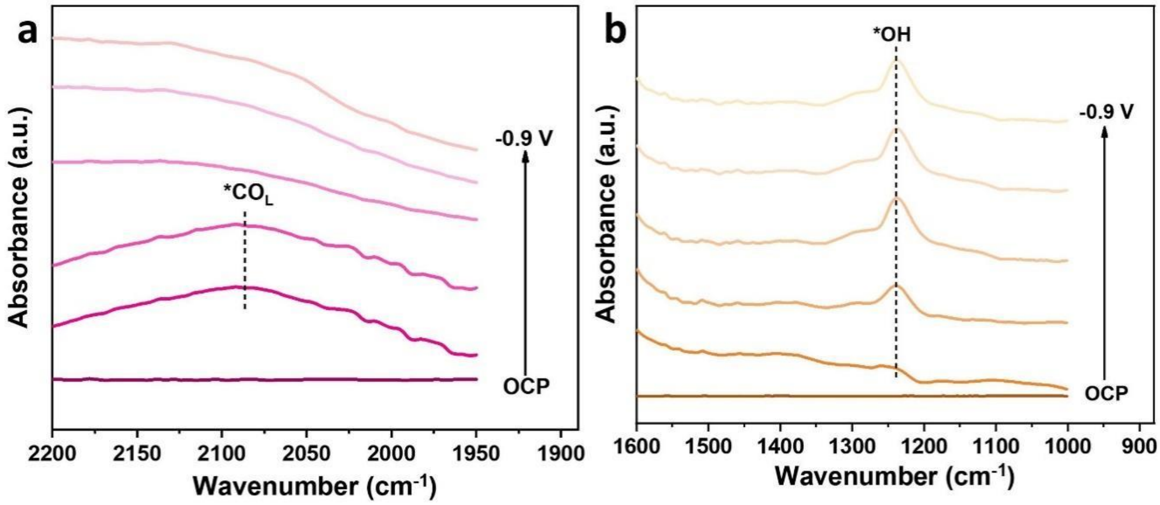

Figure S18. In-situ FTIR spectra of CO_2RR at the PtNPs as a function of the applied cathodic potential between 1950-2200 cm^{-1} (a) and 1000-1600 cm^{-1} (b).

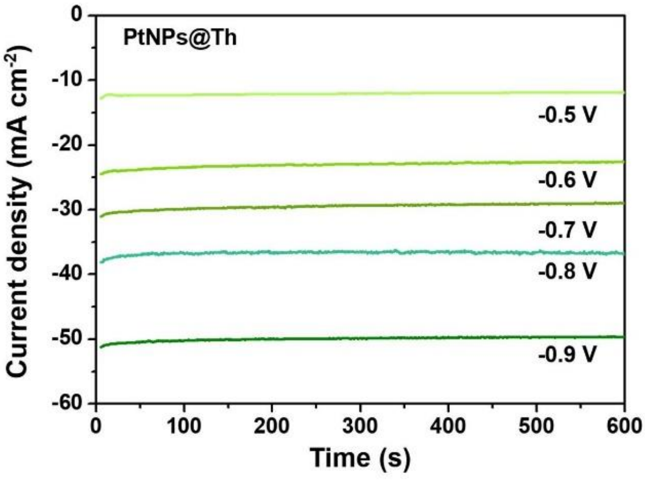

Figure S19. Current-time running of PtNPs@Th coin in different potentials under CO_2-saturated 0.5 M KCl.

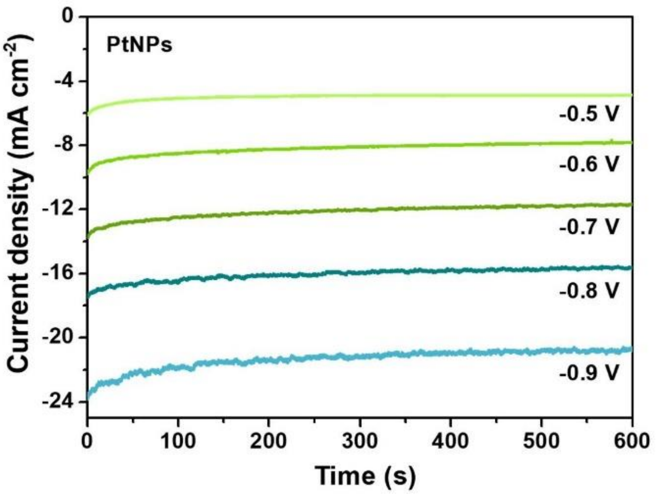

Figure S20. Current-time running of PtNPs coin in different potentials under CO_2-saturated 0.5 M KCl.

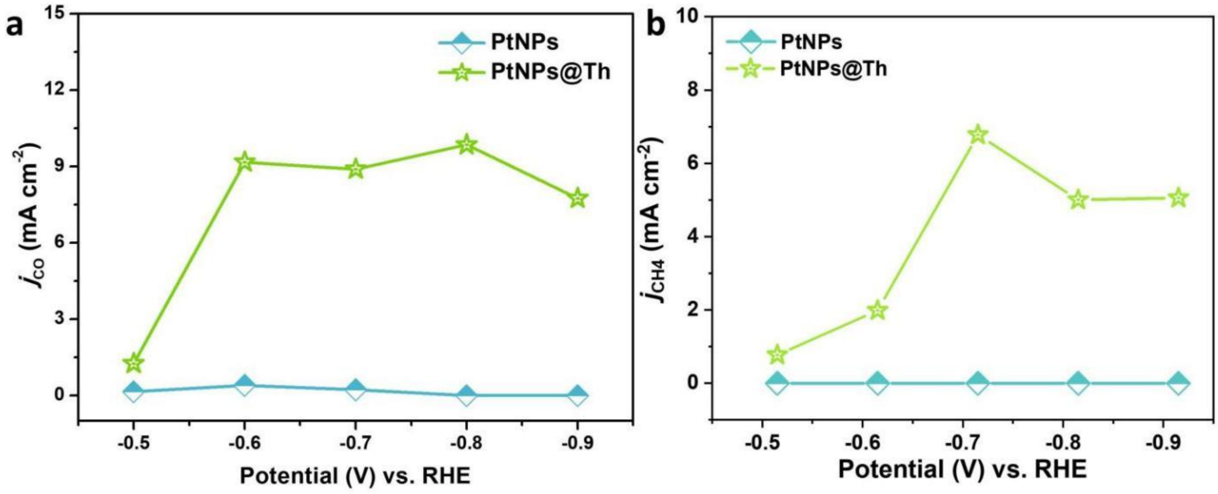

Figure S21. (a) Partial current density of CO for PtNPs and PtNPs@Th. (b) Partial current density of CH_4 for PtNPs and PtNPs@Th.

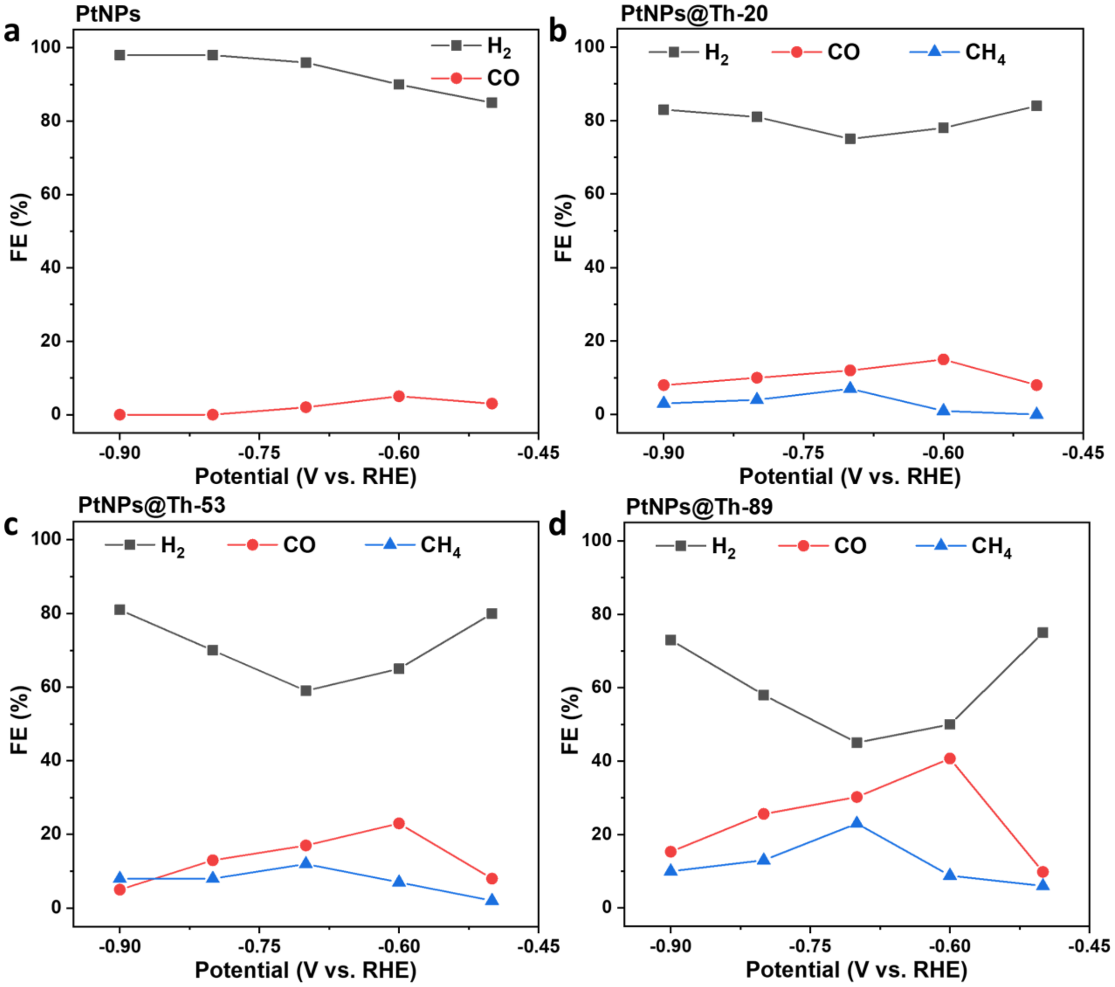

Figure S22. Faradaic efficiency of all products for PtNPs@Th encapsulated with different amounts of Th (0, 20, 53, 89%).

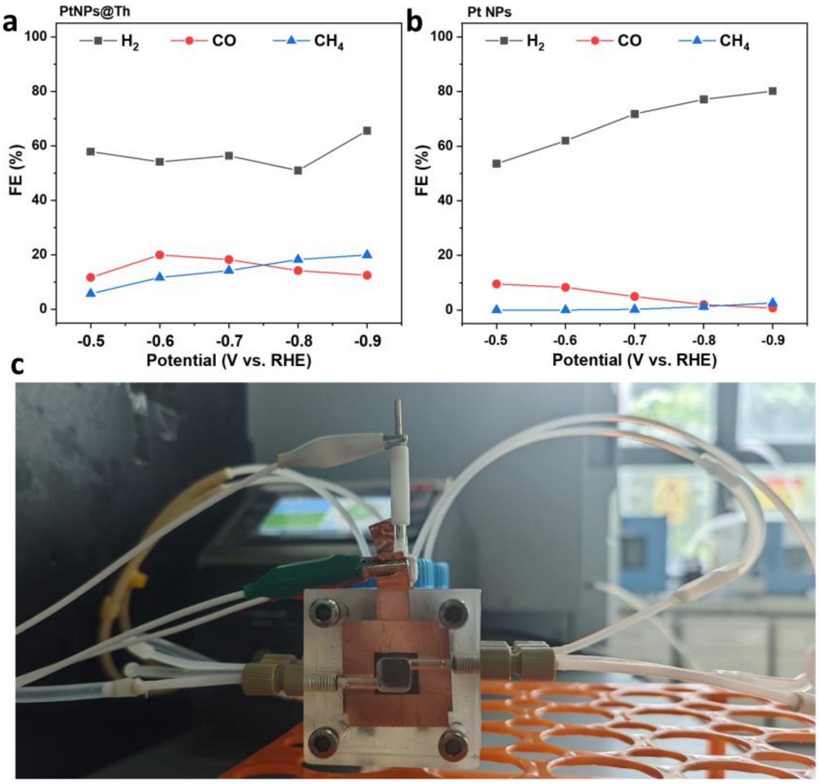

Figure S23. Faradaic efficiency of all products for (a) PtNPs@Th and (b) PtNPs@Th with gas diffusion electrode in 0.5 M KCl; (c) Diagram of electrochemical flow cell device.

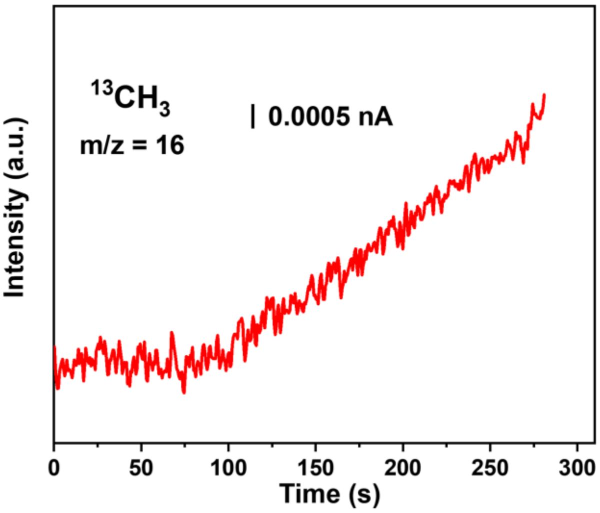

Figure S24. PtNPs@Th with the corresponding mass fragment of $^{13}CH_3$ signals of online DEMS recorded under $^{13}CO_2$ atmosphere.

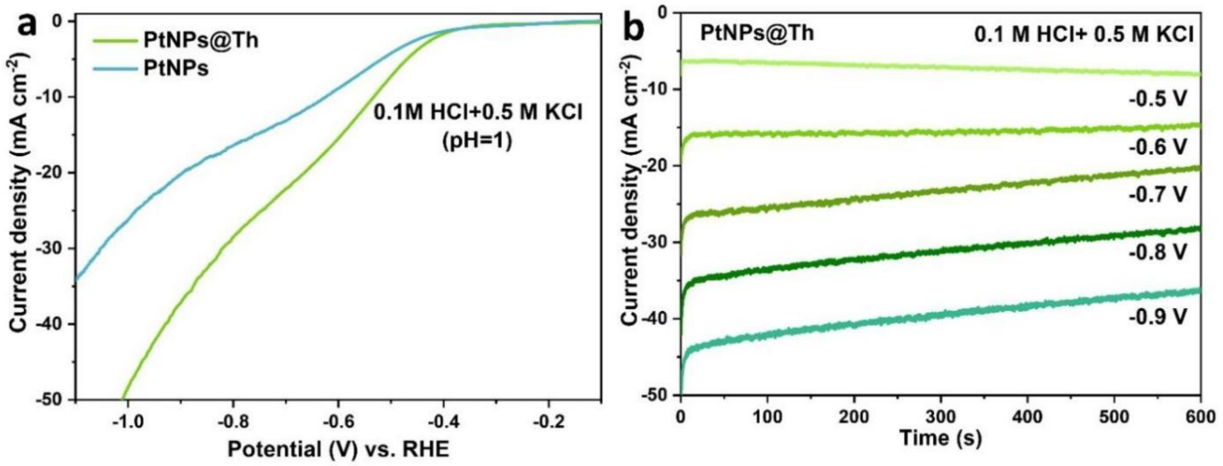

Figure S25 (a) LSV curves of PtNPs and PtNPs@Th catalysts, and (b) Current-time running of PtNPs@Th coin in different potentials under CO_2-saturated acidic electrolyte composed of 0.1 M HCl and 0.5 M KCl (pH = 1) aqueous solution.

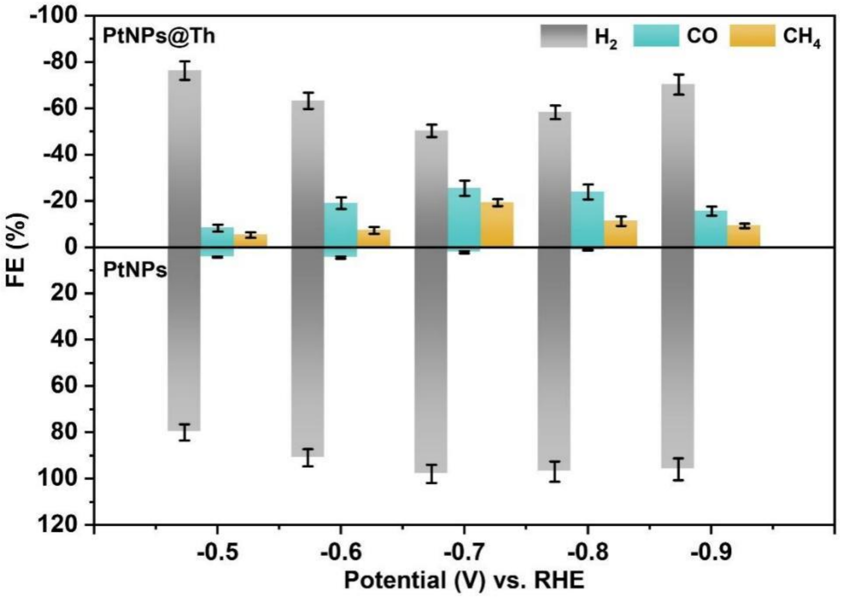

Figure S26. Faradaic efficiency of all products at PtNPs@Th and Pt NPs under CO_2-saturated acidic electrolyte composed of 0.1 M HCl and 0.5 M KCl (pH = 1) aqueous solution.

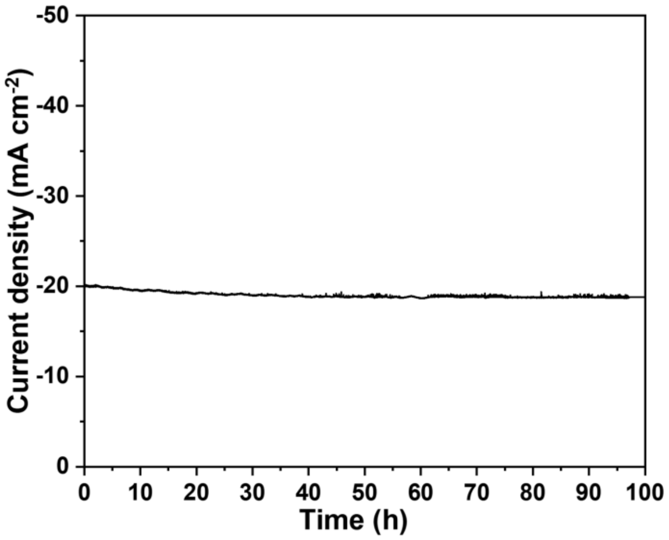

Figure S27. Long-term test of PtNPs@Th coin performed at −0.7 V vs. RHE under CO_2-saturated 0.5 M KCl (pH = 4.2) aqueous solution.

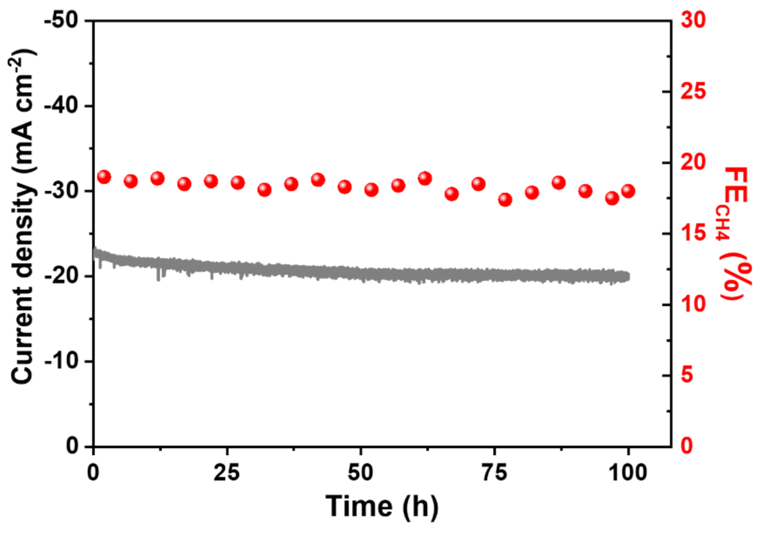

Figure S28. Long-term test of PtNPs@Th coin performed at −0.7 V vs. RHE under CO_2-saturated acidic electrolyte composed of 0.1 M HCl and 0.5 M KCl (pH = 1) aqueous solution.

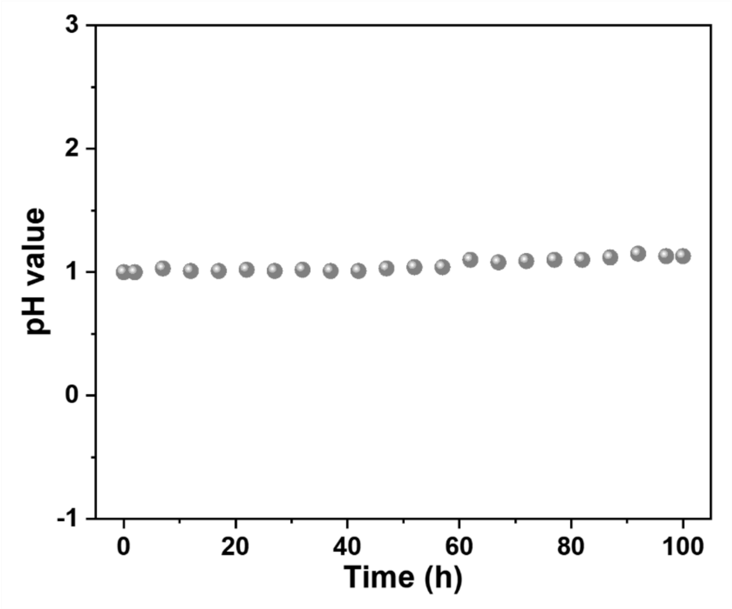

Figure S29. pH value during the long-term test of PtNPs@Th performed at −0.7 V vs. RHE under CO_2-saturated acidic electrolyte composed of 0.1 M HCl and 0.5 M KCl (pH = 1) aqueous solution.

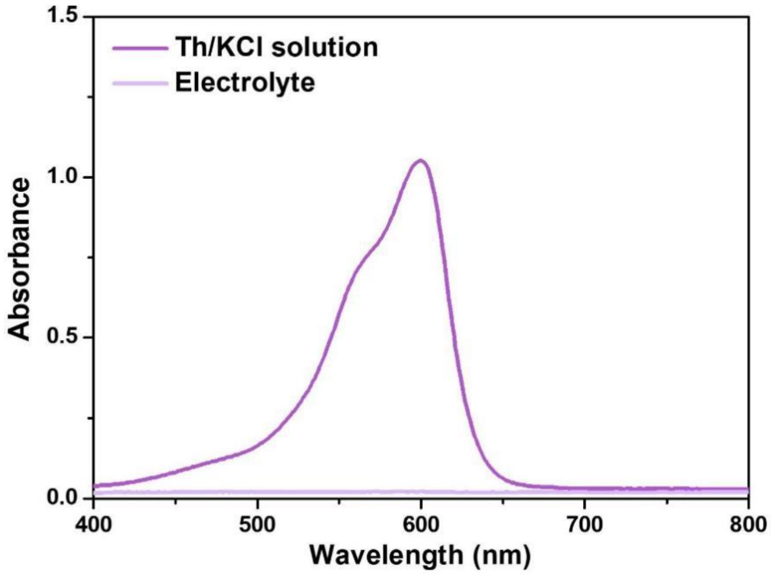

Figure S30. UV-vis spectrogram of the electrolytic solution after long-term test.

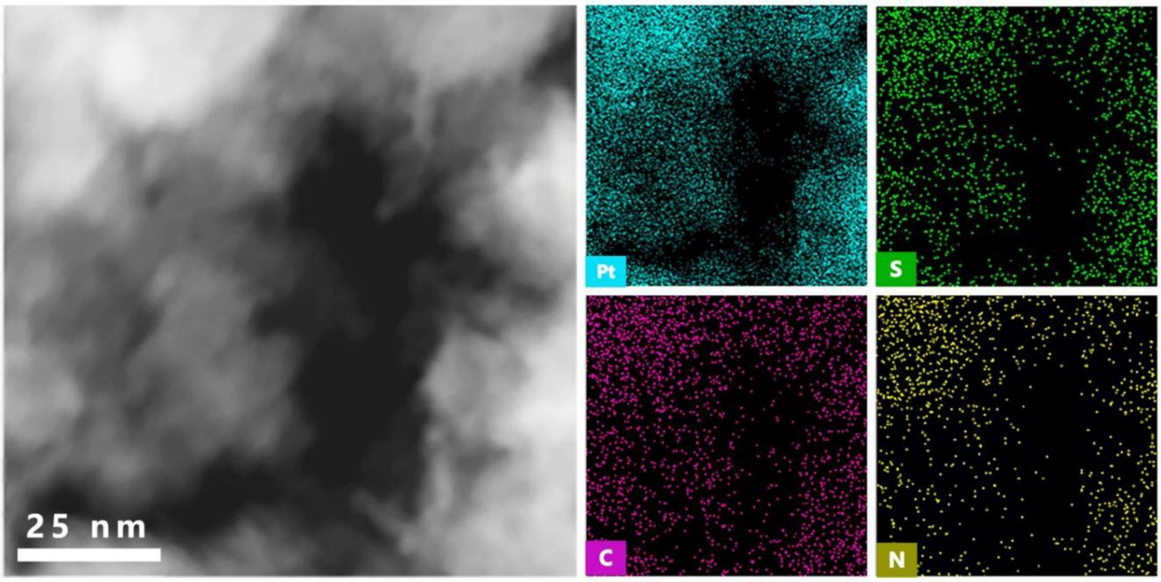

Figure S31. EDX elemental mapping images of PtNPs@Th after electrolysis, showing the signals of Pt, C, N and S.

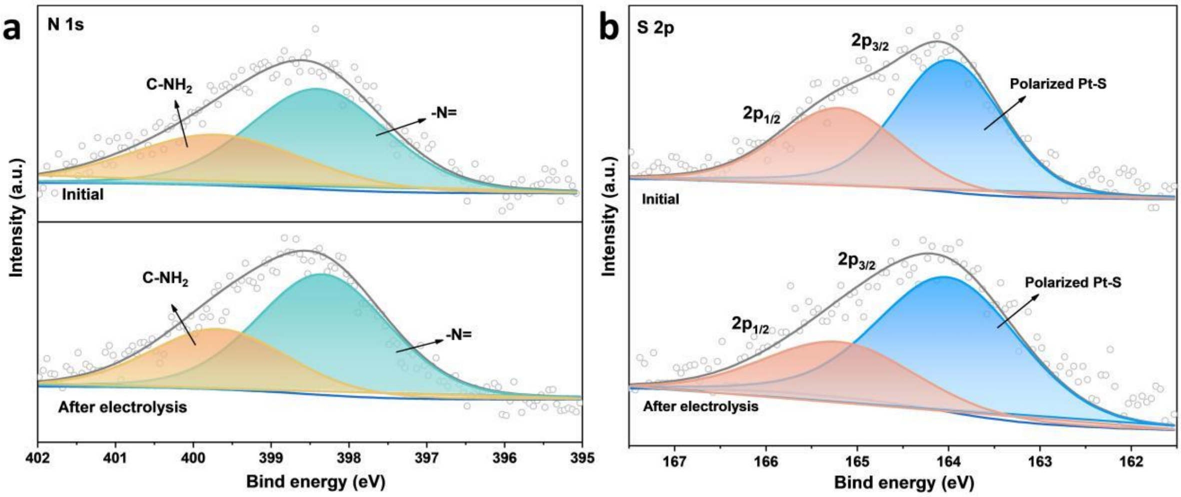

Figure S32. XPS spectra of the S element (left) and N element (right) in PtNPs@Th catalyst after electrolysis.

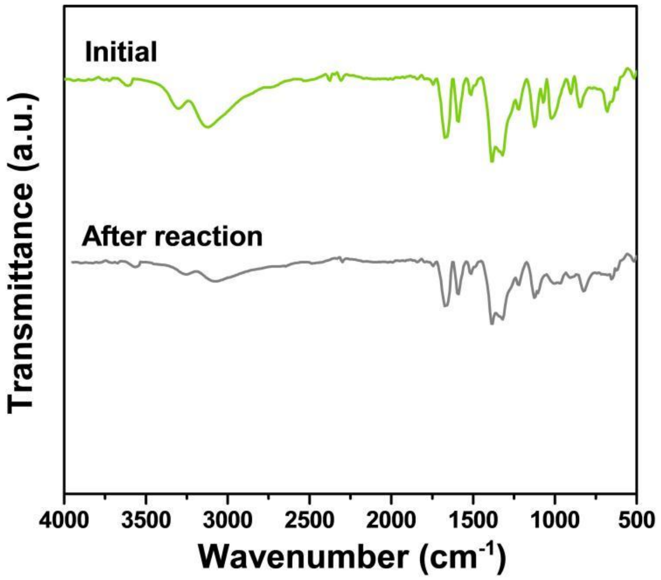

Figure S33. FT-IR spectra of PtNPs@Th catalyst before and after electrolysis.

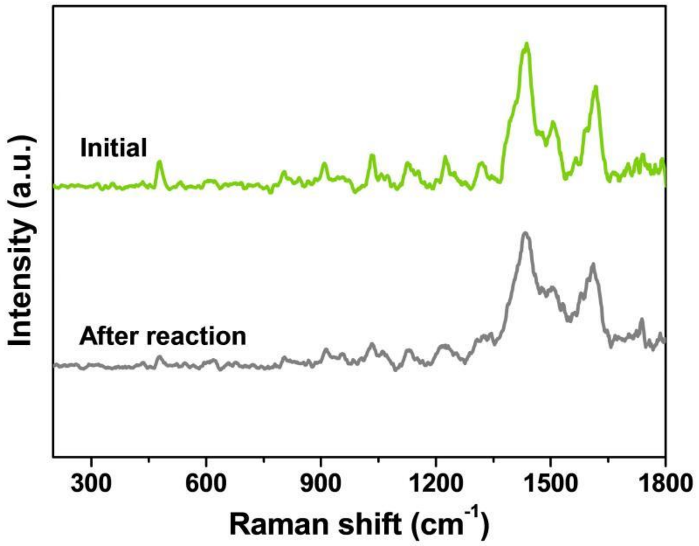

Figure S34. Raman spectra of PtNPs@Th catalyst before and after electrolysis.

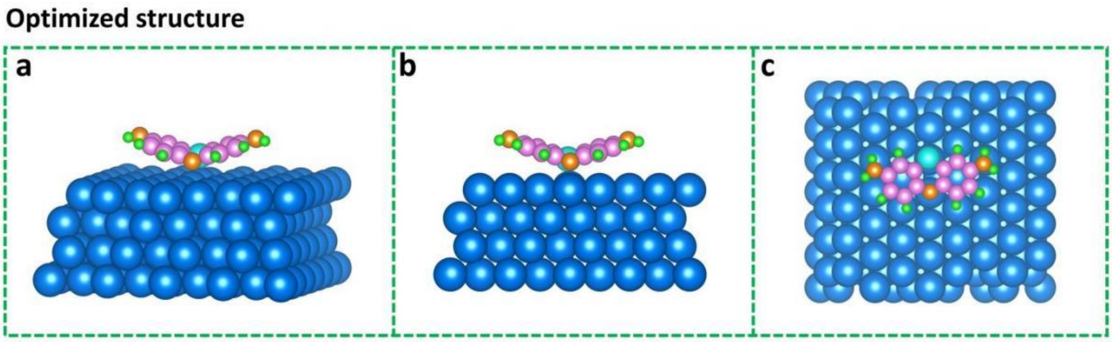

Figure S35. Optimized structure of thionine-Pt (111), (a) front view, (b) side view, (c) top view. Blue, pink, orange, cyan and green balls represent platinum, carbon, nitrogen, sulfur and hydrogen atoms, respectively.

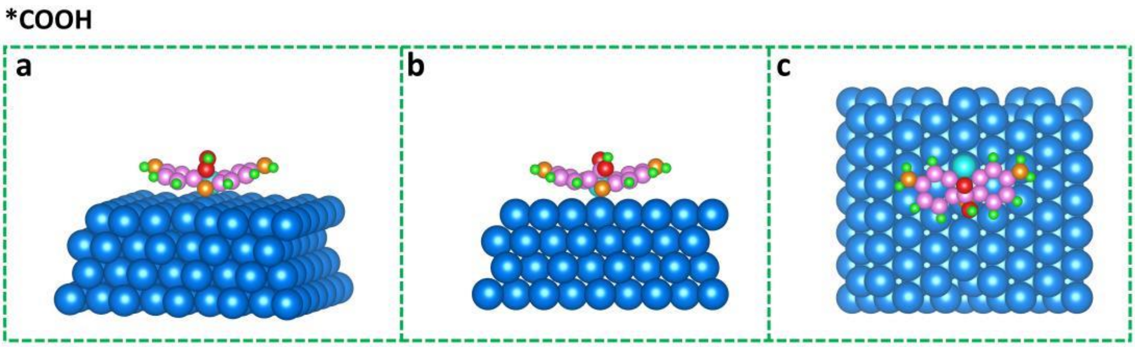

Figure S36. COOH adsorption configuration on thionine-Pt (111), (a) front view, (b) side view, (c) top view. Blue, pink, orange, cyan, red and green balls represent platinum, carbon, nitrogen, sulfur, oxygen and hydrogen atoms, respectively.

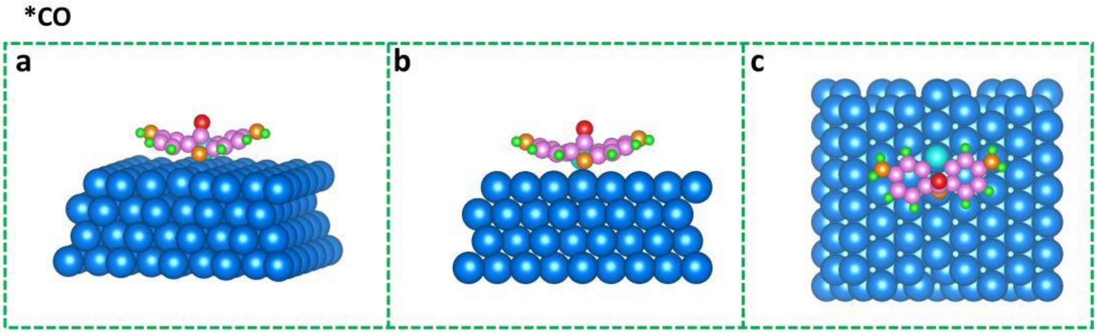

Figure S37. CO adsorption configuration on thionine-Pt (111), (a) front view, (b) side view, (c) top view. Blue, pink, orange, cyan, red and green balls represent platinum, carbon, nitrogen, sulfur, oxygen and hydrogen atoms, respectively.

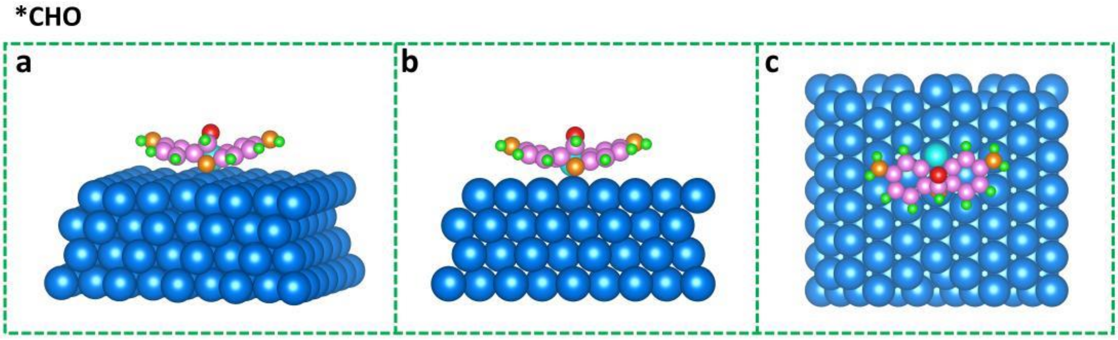

Figure S38. CHO adsorption configuration on thionine-Pt (111), (a) front view, (b) Side view, (c) top view. Blue, pink, orange, cyan, red and green balls represent platinum, carbon, nitrogen, sulfur, oxygen and hydrogen atoms, respectively.

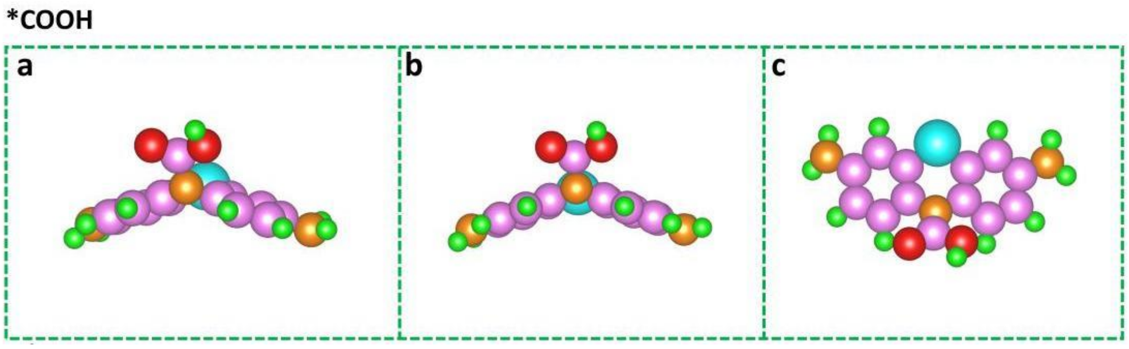

Figure S39. COOH adsorption configuration on thionine, (a) front view, (b) side view, (c) top view. Pink, orange, cyan, red and green balls represent carbon, nitrogen, sulfur, oxygen and hydrogen atoms, respectively.

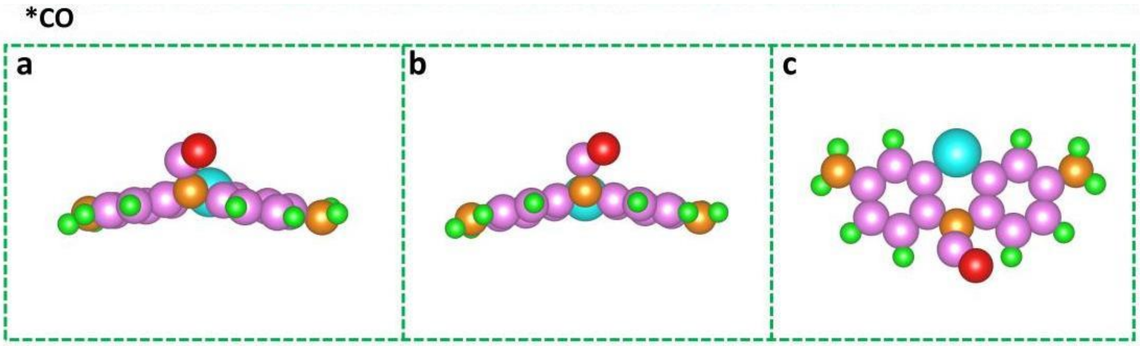

Figure S40. CO adsorption configuration on thionine, (a) front view, (b) side view, (c) top view. Pink, orange, cyan, red and green balls represent carbon, nitrogen, sulfur, oxygen and hydrogen atoms, respectively.

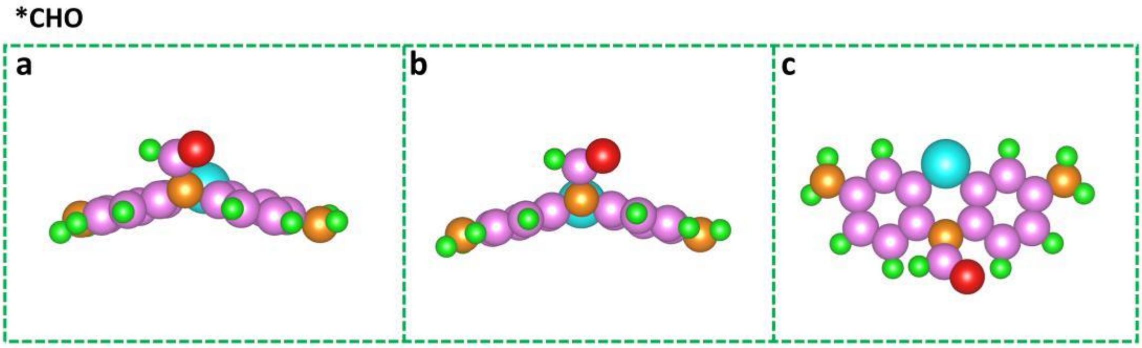

Figure S41. CHO adsorption configuration on thionine, (a) front view, (b) side view, (c) top view. Pink, orange, cyan, red and green balls represent carbon, nitrogen, sulfur, oxygen and hydrogen atoms, respectively.

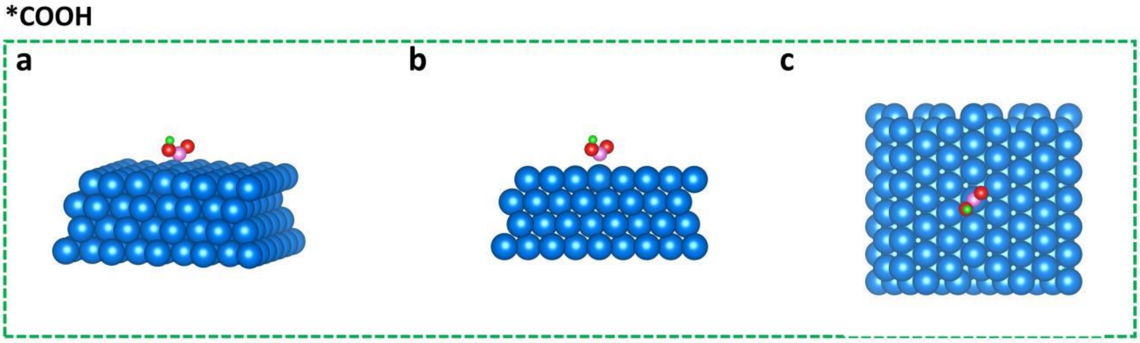

Figure S42. COOH adsorption configuration on Pt (111), (a) front view, (b) side view, (c) top view. Blue, pink, red and green balls represent platinum, carbon, oxygen and hydrogen atoms, respectively.

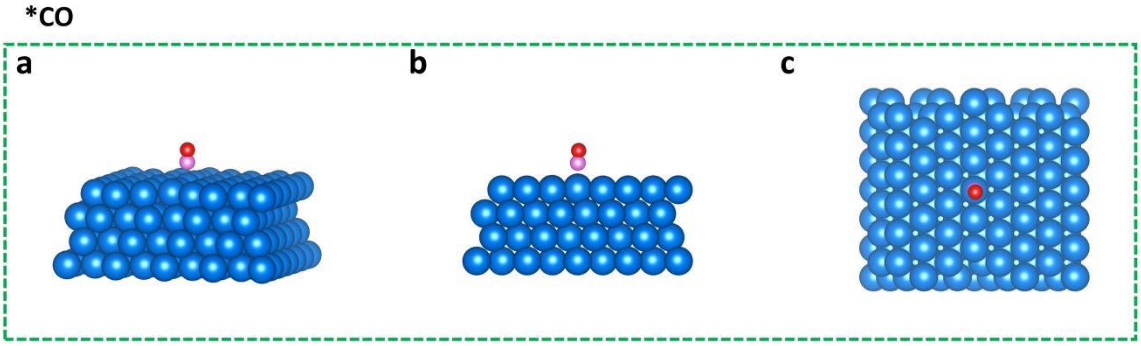

Figure S43. CO adsorption configuration on Pt (111), (a) front view, (b) side view, (c) top view. Blue, pink and red balls represent platinum, carbon and oxygen atoms, respectively.

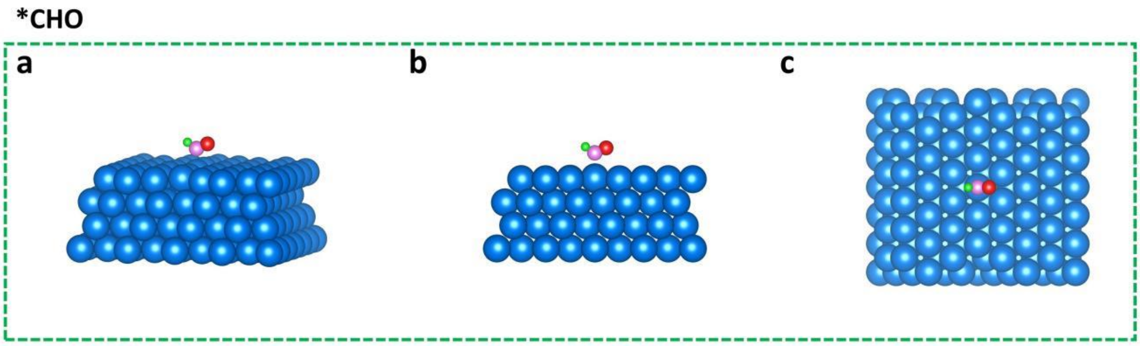

Figure S44. CHO adsorption configuration on Pt (111), (a) front view, (b) side view, (c) top view. Blue, pink, red and green balls represent platinum, carbon, oxygen and hydrogen atoms, respectively.

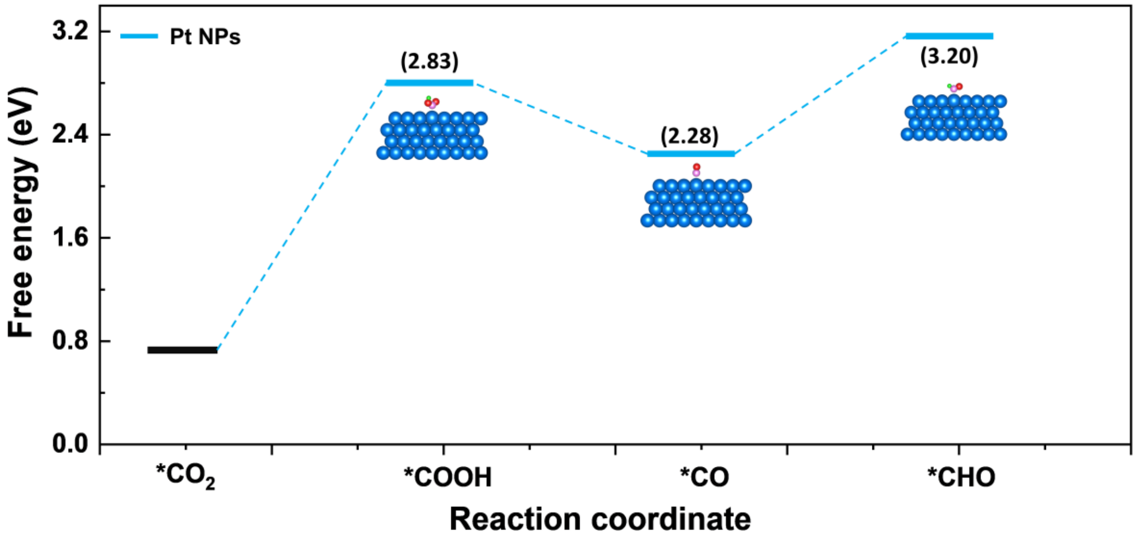

Figure S45. The calculated Gibbs free energy on Pt (111).

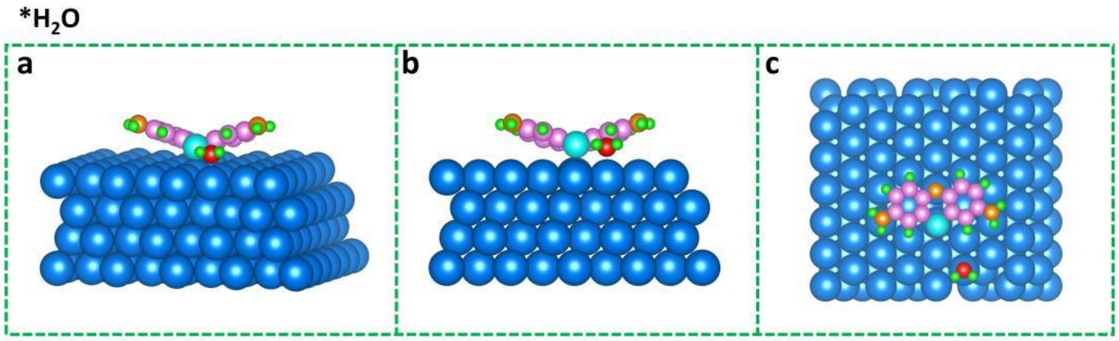

Figure S46. H_2O adsorption configuration on thionine-Pt (111), (a) front view, (b) side view, (c) top view. Blue, pink, orange, cyan, red and green balls represent platinum, carbon, nitrogen, sulfur, oxygen and hydrogen atoms, respectively.

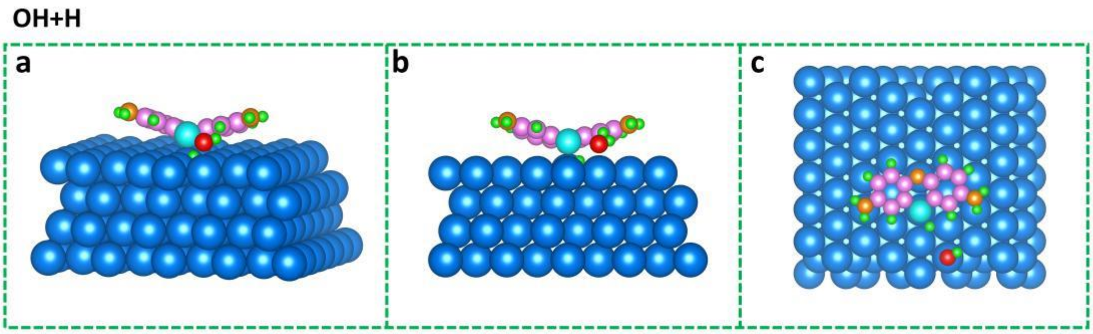

Figure S47. OH and H adsorption configuration on thionine-Pt (111), (a) front view, (b) side view, (c) top view. Blue, pink, orange, cyan, red and green balls represent platinum, carbon, nitrogen, sulfur, oxygen and hydrogen atoms, respectively.

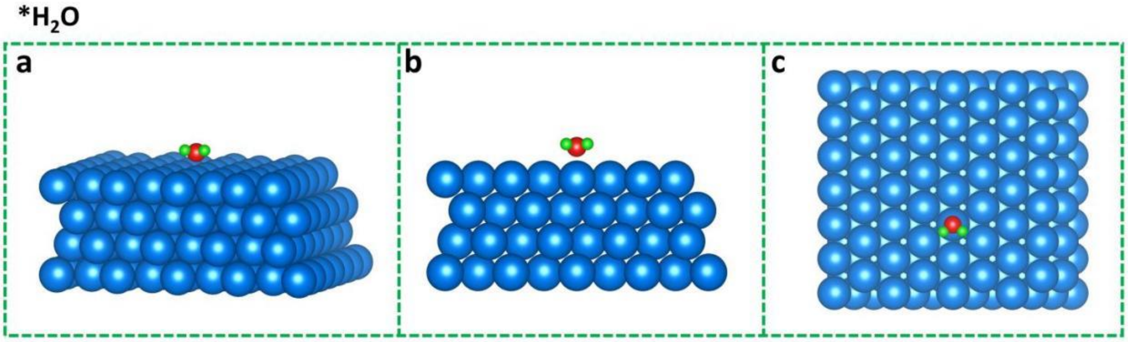

Figure S48. H_2O adsorption configuration on Pt (111), (a) front view, (b) side view, (c) top view. Blue, red and green balls represent platinum, oxygen and hydrogen atoms, respectively.

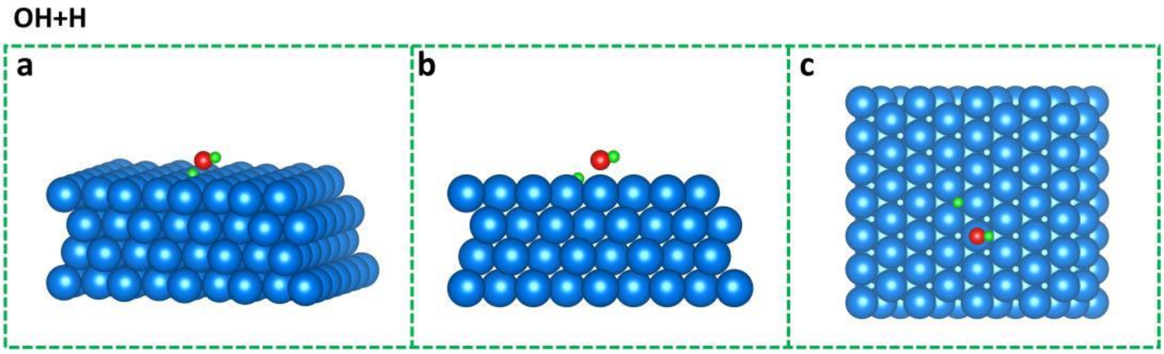

Figure S49. OH and H adsorption configurations on Pt (111), (a) front view, (b) side view, (c) top view. Blue, red and green balls represent platinum, oxygen and hydrogen atoms, respectively.

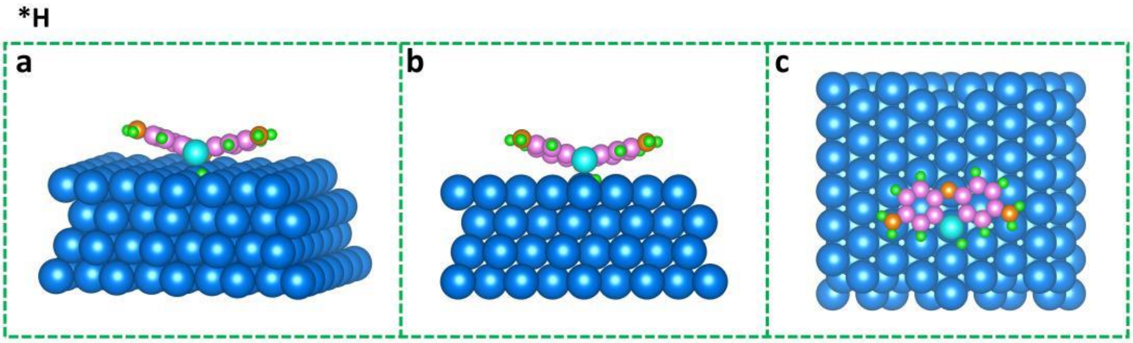

Figure S50. H adsorption configuration on thionine- Pt (111), (a) front view, (b) side view, (c) top view. Blue, pink, orange, cyan, and green balls represent platinum, carbon, nitrogen, sulfur, and hydrogen atoms, respectively.

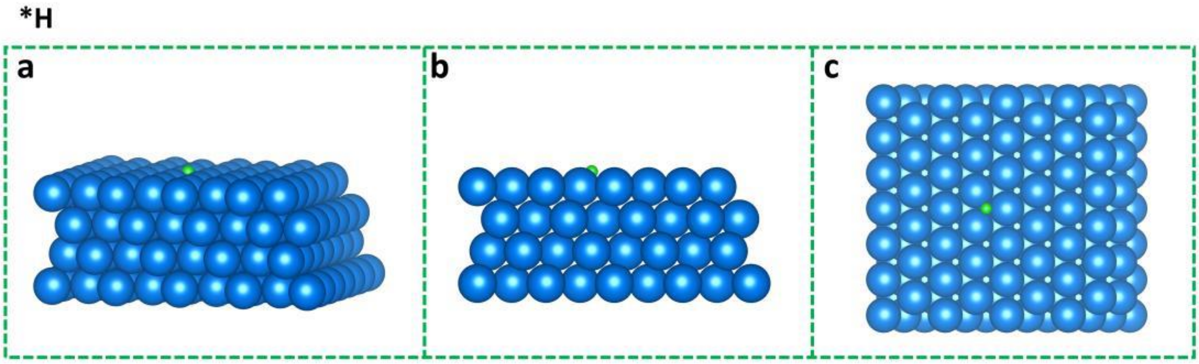

Figure S51. H adsorption configuration on Pt (111), (a) front view, (b) side view, (c) top view. Blue and green balls represent platinum and hydrogen atoms, respectively.

Table S1 Summary of entrapment value and Th loading amount for preparing PtNPs@Th-X samples.

Sample	starting Th/Pt molar ratio	entrapment of Th (%)	wt% ratio (Th/sample)
PtNPs@Th	1:300	89%	1.5
PtNPs@Th-53	1:500	53%	0.9
PtNPs@Th-20	1:700	20%	0.5
PtNPs	0	0	0

Table S2 The performance comparison of electrocatalytic CO_2 methanation of PtNPs@Th with other electrocatalysts.

Electrocatalyst	Electrolyte	FE_{CH4} (%)	Production rate (mmol h^{-1} cm^{-2})	pH	Ref.
Cu nanoparticle	0.1 M $KHCO_3$	12.1	1.6E-3	---	[9]
Cu-18-6-C	0.5M K_2SO_4+H_2SO_4	51.19	1.43	2	[10]
Cu/PTFE	1 M $KHCO_3$	48	0.8	8.5	[11]
Cu/CeO_2-350	1 M KOH	42	0.24	14	[12]
Pt/C	0.5 M H_2SO_4	3	---	---	[13]
Pt/C	0.5 M H_2SO_4	6.8	---	---	[14]
Pt-black	0.5 M H_2SO_4	23.2	---	---	[15]
PtNPs@Th	0.5 M KCl	23%	1.3	4.2	This work
	0.5 M KCl+0.1 M HCl	19%	2.3	1	This work

Supplementary Movies

Movie S1. The stability test of PtNPs@Th composite in H_2O and dimethyl sulfoxide (DMSO).

Movie S2. The stability test of PtNPs@Th cathode in long-term electrolysis.

Supplementary References

[1] Kresse, G.; Joubert, D. From ultrasoft pseudopotentials to the projector augmented-wave method. *Phys. Rev. B* **1999**, *59*, 1758-1775.

[2] Blöchl, P. E. Projector augmented-wave method. *Phys. Rev. B* **1994**, *50*, 17953-17979.

[3] Perdew, J. P.; Wang, Y. Accurate and simple analytic representation of the electron-gas correlation energy. *Phys. Rev. B* **1992**, *45*, 13244-13249.

[4] Grimme, S.; Antony, J.; Ehrlich, S.; Krieg, H. A consistent and accurate ab initio parametrization of density functional dispersion correction (DFT-D) for the 94 elements H-Pu. *J. Chem. Phys*. **2010**, *132*, 154104.

[5] Wang, X.; Zhou, H.; Yan, Z.; Zhang, X.; Jia, J.; Wu, H. Boron-/Fe-codoped graphene as high-activity single-atom catalyst. *Theor. Chem. Acc*. **2017**, *136*, 1-6.

[6] Nørskov, J. K.; Bligaard, T.; Logadottir, A.; Kitchin, J. R.; Chen, J. G.; Pandelov, S.; Stimming, U., Trends in the exchange current for hydrogen evolution. *J. Electrochem. Soc*. **2005**, *152*, J23-J26.

[7] Yan, Y.; Zeitler, E. L.; Gu, J.; Hu, Y.; Bocarsly, A. B., Electrochemistry of aqueous pyridinium: Exploration of a key aspect of electrocatalytic reduction of CO_2 to methanol. *J. Am. Chem. Soc.* **2013**, *135*, 14020-14023.

[8] Dai, J.; Zhu, Y.; Chen, Y.; Wen, X.; Long, M.; Wu, X.; Hu, Z.; Guan, D.; Wang, X.; Zhou, C.; Lin, Q.; Sun, Y.; Weng, S.-C.; Wang, H.; Zhou, W.; Shao, Z., Hydrogen spillover in complex oxide multifunctional sites improves acidic hydrogen evolution electrocatalysis. *Nat. Commun*. **2022**, 13, 1189.

[9] Merino-Garcia, I.; Albo, J.; Irabien, A., Productivity and Selectivity of Gas-Phase CO_2 Electroreduction to Methane at Copper Nanoparticle-Based Electrodes. *Energy Technol.* **2017**, *5*, 922-928.

[10] Xu, K.; Li, J.; Liu, F.; Chen, X.; Zhao, T.; Cheng, F., Favoring CO intermediate

stabilization and protonation by crown ether for CO_2 electromethanation in acidic media. *Angew. Chem. Int. Ed.* **2023**, *135*, e202311968.

[11] Wang, X.; Xu, A.; Li, F.; Hung, S.-F.; Nam, D.-H.; Gabardo, C. M.; Wang, Z.; Xu, Y.; Ozden, A.; Rasouli, A. S.; Ip, A. H.; Sinton, D.; Sargent, E. H., Efficient methane electrosynthesis enabled by tuning local CO_2 availability. *J. Am. Chem. Soc.* **2020**, *142*, 3525-3531.

[12] Patra, K. K.; Liu, Z.; Lee, H.; Hong, S.; Song, H.; Abbas, H. G.; Kwon, Y.; Ringe, S.; Oh, J., Boosting electrochemical CO_2 reduction to methane via tuning oxygen vacancy concentration and surface termination on a copper/ceria catalyst. *ACS Catal.* **2022**, *12*, 10973-10983.

[13] Matsuda, S.; Tamura, S.; Yamanaka, S.; Niitsuma, Y.; Sone, Y.; Umeda, M., Minimization of Pt-electrocatalyst deactivation in CO_2 reduction using a polymer electrolyte cell. *React. Chem. Eng.* **2020**, *5*, 1064-1070.

[14] Umeda, M.; Niitsuma, Y.; Horikawa, T.; Matsuda, S.; Osawa, M., Electrochemical reduction of CO_2 to methane on platinum catalysts without overpotentials: Strategies for improving conversion efficiency. *ACS Appl. Energy Mater.* **2019**, *3*, 1119-1127.

[15] Matsuda, S.; Yoshida, Y.; Umeda, M., Electroreduction of CO_2 to CH_4 without overpotential using Pt-black catalysts: Enhancement of faradaic efficiency. *Int. J. Energy Res.* **2022**, *46*, 9919-9925.
